# IL4I1: a novel molecular biomarker represents an inflamed tumor microenvironment and precisely predicts the molecular subtype and immunotherapy response of bladder cancer

**DOI:** 10.3389/fphar.2024.1365683

**Published:** 2024-05-30

**Authors:** Xiangrong Peng, Chuan Liu, Li Zhang, Yin Chen, Lixin Mao, Shenglin Gao, Xiaokai Shi, Li Zuo

**Affiliations:** ^1^ Department of Urology, ChangZhou No.2 people’s Hospital, Nanjing Medical University, Changzhou, Jiangsu, China; ^2^ Laboratory of Urology, ChangZhou Medical Center, Nanjing Medical University, Changzhou, Jiangsu, China; ^3^ Department of Urology, Gonghe County Hospital of Traditional Chinese Medicine, Hainan Tibetan Autonomous Prefecture, Qinghai, China

**Keywords:** bladder cancer, inflamed tumor microenvironment, IL4I1, immunotherapy, molecular subtype

## Abstract

**Introduction:** IL4I1, also known as Interleukin-4-induced gene 1, is an enzyme that can modulate the immune system by acting as a L-amino acid oxidase. Nevertheless, a precise understanding of the correlation of IL4I1 with immunological features and immunotherapy efficacy in bladder cancer (BLCA) remains incomplete.

**Methods:** We analyzed RNA sequencing data from the Cancer Genome Atlas (TCGA) to investigate the immune function and prognostic importance of IL4I1 across different cancer types. We further examined the TCGA-BLCA cohort for correlations between IL4I1 and various immunological characteristics of tumor microenvironment (TME), such as cancer immune cycle, immune cell infiltration, immune checkpoint expression and T cell inflamed score. Validation was conducted using two independent cohort, GSE48075 and E-MTAB-4321. Finally, RNA sequencing data from the IMvigor210 cohort and immunohistochemistry assays were employed to validate the predictive value of IL4I1 for the TME and immunotherapy efficacy.

**Results:** In our findings, a positive correlation was observed between IL4I1 expression and immunomodulators expression, immune cell infiltration, the cancer immune cycle, and T cell inflamed score in BLCA, suggesting a significant link to the inflamed TME. In addition, studies have shown that IL4I1 elevated levels of individuals tend to be more performance for basal subtype and exhibit enhanced response rates to diverse treatment modalities, specifically immunotherapy. Clinical data from the IMvigor 210 cohort confirmed a higher rate of response to immunotherapy and better survival benefits in patients with high IL4I1 expression.

**Discussion:** To summarize, our research showed that elevated IL4I1 levels are indicative of an inflamed TME, the basal subtype, and a more favorable response to various treatment methods, especially immune checkpoint blockade therapy in BLCA.

## Introduction

Bladder cancer (BLCA) is among the prevalent urological cancers globally. By 2023, BLCA ranked seventh in incidence among all malignant tumors and fourth in incidence among men globally (Siegel et al., 2023). Surgical removal is the primary approach for initial instances of BLCA, and the outlook for advanced metastatic BLCA remains unfavorable despite the utilization of neoadjuvant and adjuvant chemotherapy (Witjes et al., 2021). In the past few years, immunotherapy, particularly immune checkpoint blockade (ICB) treatment involving anti-PD-1/PD-L1, has provided hopeful survival advantages for individuals with advanced BLCA, and has greatly enhanced the treatment condition for those with advanced BLCA (Necchi et al., 2017; Powles et al., 2018). Nevertheless, due to the presence of either primary or secondary resistance mechanisms, the ICB proves to be effective for only a minority of patients (Rosenberg et al., 2016; Sharma et al., 2017). This suggests that there are variations in the immune status of each host during cancer development. The effective application of ICB therapy heavily relies on the presence of anti-cancer immune response and an inflamed tumor microenvironment (TME) in patients (Ji et al., 2012; Chen and Mellman, 2017). The TME contains a diverse combination of cells, comprising both tumor cells and non-tumor cells. The two major constituents among the non-tumor cells are immune cells and stromal cells (Eruslanov et al., 2012; Müller et al., 2012; Said and Theodorescu, 2012). The levels and spatial arrangement of tumor infiltrating lymphocytes (TILs) have the potential to indicate tumor inflammation stages, subtypes, and patient survival rates. Elevated levels of TILs are indicative of an inflamed subtype, which is associated with a disease-specific 5-year survival rate of 80%, while the absence of immune infiltration is considered a non-inflamed subtype with the survival rate of below 25% (Pfannstiel et al., 2019). Therefore, we need biomarkers to define TME subtypes in order to predict the effectiveness of immunotherapy.

The antigen processing and presentation by tumor cells and immune cells plays a pivotal role in the activation of T cells and the generation of a long-lasting clinical response to ICIs. Integration of the antigen presentation machinery (APM), molecular and clinical data have demonstrated the ability to predict the efficacy of immunotherapy (Li et al., 2021; Yang et al., 2023). Cytotoxic T lymphocytes (CTL) kill cancer cells by releasing granules or inducing FasL-mediated apoptosis. However, due to immunosuppressive interactions between tumor cells and stromal cells, the function of CTL is suppressed. Increasing research had shown that the infiltration levels of CTL influence the efficacy of immune checkpoint blockade (ICB) therapy (SNYDER et al., 2014; Farhood et al., 2019). Additionally, IFN-γ is a key cytokine for activated T cells as well as natural killer (NK) and NK T cell production in the tumor microenvironment (Ikeda et al., 2002). And IFN-γ signaling enables the PD-1 signaling axis to become activated to downregulate the cytotoxic response (Abiko et al., 2015; Bellucci et al., 2015; Ayers et al., 2017). These are considered be potential biomarkers to predict clinical response to immunotherapy. In addition to, various biomarkers are also used to employe for the prediction of effectiveness, which encompass PD-L1 mRNA levels, tumor mutation burden (TMB), microsatellite instability (MSI), and BLCA molecular subtypes (McLaughlin et al., 2016; Bonneville et al., 2017; Kamoun et al., 2020; Sha et al., 2020). However, they also have limitations. Various factors can affect the accuracy of PD-L1 expression prediction, while the complex, slow, and expensive nature of identifying TMB, MSI, and molecular subtypes limits their clinical usability (McLaughlin et al., 2016; Bonneville et al., 2017; Nishino et al., 2017; Kamoun et al., 2020; Sha et al., 2020). Therefore, there is an immediate requirement to investigate novel biomarkers that are stable, convenient, and cost-effective.

IL4I1, a gene induced by Interleukin-4 (IL-4), was first discovered in B spleen cells of mice after being stimulated by IL-4 (Chu and Paul, 1997). IL4I1 exhibits L-amino acid oxidase activity and predominantly metabolizes L-phenylalanine, with a minor metabolic involvement in L-arginine, at the physiologically optimal pH (Boulland et al., 2007). IL4I1 has been described to be expressed primarily within the human immune system, central nervous system, and sperm, where it regulates immune cell differentiation and activation, affects sperm function, or promotes central nervous system development (Chavan et al., 2002; Houston et al., 2015; Pluchino and Peruzzotti-Jametti, 2016). Moreover, IL4I1 is closely linked to tumor progression. It is considered a metabolic immune checkpoint, and IL4I1-mediated catabolism of tryptophan (Trp) produces indoles and kynurenic acid, which activate aromatic receptors (AHR), thereby promoting cancer cell movement and inhibiting adaptive immunity (Sadik et al., 2020; Castellano et al., 2021). Excessive expression of IL4I1 has been detected in primary mediastinal B cell lymphoma, where it functions as a regulator of immune response by suppressing the proliferation of T lymphocytes (Boulland et al., 2007). A vitro experiments showed that the suppression IL4I1 led to the hindrance of ovarian cancer cell growth, movement, and infiltration (Zhao et al., 2021). IL4I1 has the ability to decrease the activity of CD8^+^ T cells, enhance the development of inducible regulatory (iTreg) cells, and limit the expansion of T helper 17 (Th17) cells. In addition to promoting tumor evasion, it also minimizes the potentially detrimental impact of adaptive immune responses in chronic inflammatory conditions (Romagnani, 2016). These studies indicate the close association of IL4I1 with immune regulation and its ability to modulate the TME, thereby influencing tumor occurrence and progression. However, the complete understanding of IL4I1’s role in BLCA remains to be fully clarified.

We extensively investigated the immunological characteristics of IL4I1 in BLCA through the multiple cohort analysis in our research. The study findings emphasize the robust correlation between the IL4I1 expression level and TME in BLCA, showcasing its accurate prognostic potential for BLCA molecular subtypes, inflamed TME, and response to immunotherapy. The results offer crucial hints for a more profound understanding of the immunological traits of BLCA and provide valuable perspectives for the development of personalized immunotherapeutic strategies.

## Materials and methods

**FIGURE 1:**
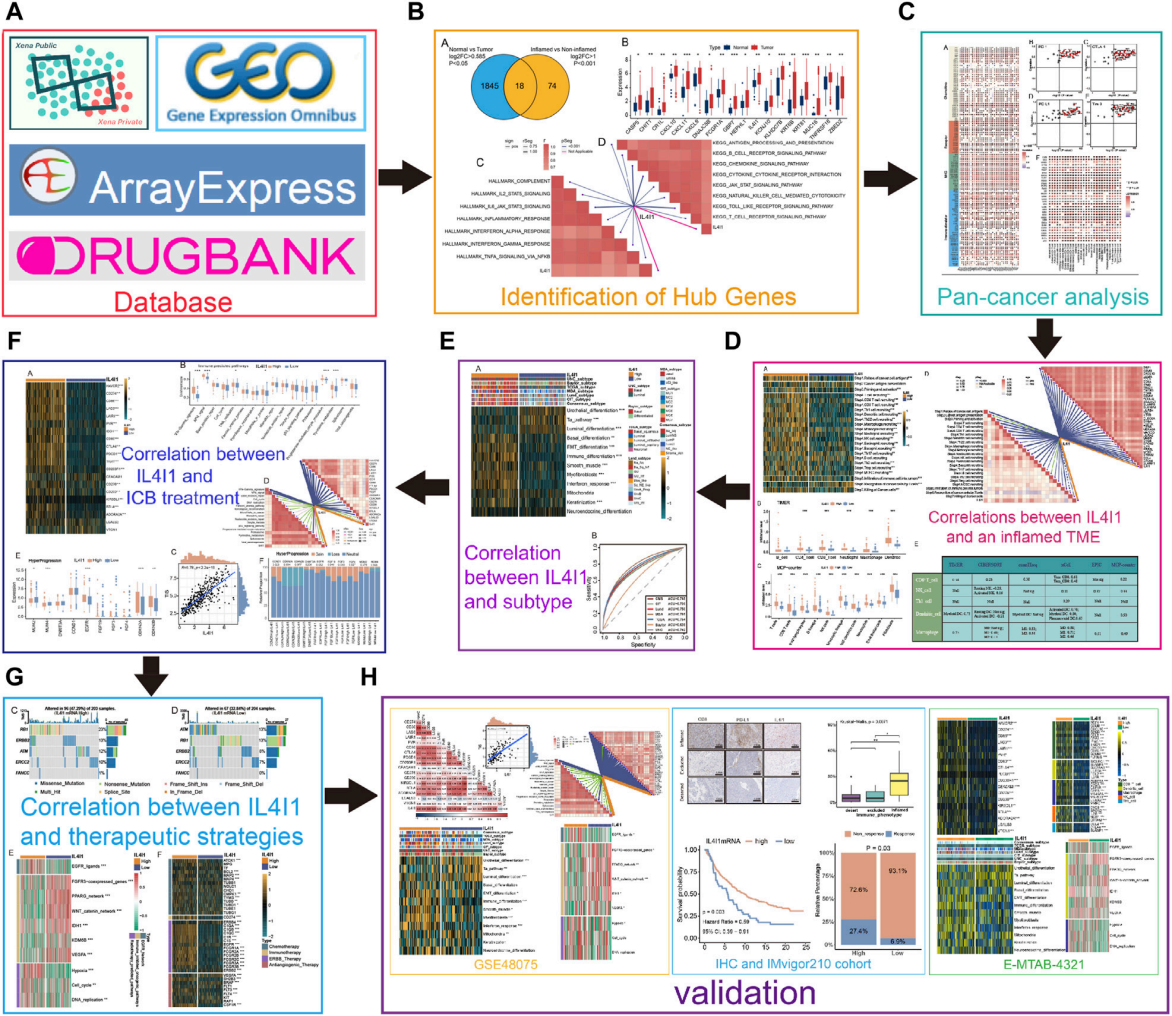
An overview of designation in our study. **(A)** The database used in this study. **(B)** Identifying differential expressed genes (DEGs) for inflamed and non-inflamed phenotypes. **(C)** Correlations between IL4I1 and pan-cancer immunological features. **(D)** Correlations between IL4I1 and an inflamed TME. **(E)** The predictive value of IL4I1 for the molecular subtypes in BLCA. **(F)** The predictive value of IL4I1 for clinical response of ICB treatment. **(G)** Correlation between IL4I1 and therapeutic strategies. **(H)** Validation by external cohorts and immunohistochemistry.

The flowchart of this study is demonstrated in Figure 1.

### Data acquisition and preprocessing

The RNA sequencing data (FPKM value), somatic mutation data, and clinicopathological features for various cancer types of The Cancer Genome Atlas (TCGA) were obtained from the UCSC Xena (https://xenabrowser.net/datapages/) data portal (Goldman et al., 2020). FPKM values were converted into TPM values using the following formula:

$${TPM}_{i}=\frac{FPKM_{i}}{\Sigma_{j}FPKM_{j}}\times 10^{6}$$

Following that, we applied a log2^(TPM+1)^ transformation to the TPM value for further investigation. The somatic mutation data were processes via Varscan (https://varscan.sourceforge.net/) (Koboldt et al., 2013). The data on copy number variation (CNV) of the genes that indicate hyperprogression were obtained from a previous research (Hu et al., 2021). Additionally, for external validation purposes, we downloaded three independent cohorts namely GSE48075,E-MTAB-4321 and IMvigor210 (http://research-pub.gene.com/IMvigor210CoreBiologies/) (Choi et al., 2014; Hedegaard et al., 2016; Mariathasan et al., 2018). And we collected six immunotherapy cohorts in melanoma. These datasets were also converted from FPKM to TPM values for subsequent analysis.

### Exploration of the immunological characteristics of the TME in BLCA

The immunological features of TME in BLCA comprise the presence of immunomodulators, the functioning of the cancer immunity cycle, the degree of infiltration by tumor infiltrating immune cells (TIICs), and the presence of inhibitory immune checkpoints. Initially, data on 121 immunomodulators, encompassing major histocompatibility complex (MHC), receptors, chemokines, and immunostimulators, were gathered from Charoentong et al.'s research (Charoentong et al., 2017). The cancer immunity cycle represents the immune response against cancer and comprises of seven stages: release of cancer cell antigens (step 1), cancer antigen presentation (step 2), priming and activation (step 3), trafficking of T cells to tumors (step 4), infiltration of T cells into tumors (step 5), recognition of cancer cells by T cells (step 6), and killing of cancer cells (step 7) (Chen and Mellman, 2013). Investigation of these steps' activity was conducted through the utilization of a single sample gene set enrichment analysis (ssGSEA).

Using the R package “GSVA,” we performed ssGSEA to assess the extent of TIIC infiltration in the BLCA, utilizing signatures from the TISIDB database (Ru et al., 2019). Then, to avoid bias, six independent algorithms were used to determine the level of TIIC infiltration in BLCA, namely, TIMER (Li et al., 2017), MCP-counter (Becht et al., 2016), CIBERSORT (Newman et al., 2015), quanTIseq (Finotello et al., 2019), xCell (Aran et al., 2017), and EPIC (Racle et al., 2017). From previous studies, we adopted effector genes of TIICs as well (Hu et al., 2021). Next, we gathered several inhibitory immune checkpoints with promising therapeutic potential as reported in Auslander’s research (Auslander et al., 2018).

T cell inflammation score (TIS), was employed for assessing the inflammation level within the TME in BLCA. TIS serves as a previously developed predictive factor for assessing the effectiveness of cancer immunotherapy and anti-PD-1 therapy (Ayers et al., 2017). Calculating the TIS, which includes 18 IFN-γ-responsive genes, helps to reflect pre-existing anti-cancer immunity and predict the clinical response to ICB. Genes collected in this study were identified by Ayers et al. (Ayers et al., 2017).

$$TIS=\Sigma_{\gamma =1}^{18}\beta_{\gamma }X_{\gamma }$$

where βγ represents the coefficient of a gene predefined in a previous study, and Xγ is the expression level of this gene.

Hyperprogression is considered to be an adverse event of abnormally accelerated tumor growth when ICB is performed. We identified several predictors used to predict hyperprogression from previous studies (Kato et al., 2017; Singavi et al., 2017; Giusti et al., 2019). Kato et al. reported in Clinical Cancer Research on a study investigating the association between genomic variants and hyperprogression. Among 155 patients with tumors treated with anti-PD-1/PD-L1 monotherapy, the study revealed that six patients with MDM2/MDM4 and DNMT3A amplification experienced TTF of less than 2 months. Singavi found that patients with chromosome 11 region 13 amplification variants (CCND1, FGF3, FGF4, and FGF19 amplification) were prone to hyperprogression on immunotherapy by examining the occurrence of somatic mutations in 696 patients with solid tumors. Giusti et al. collected clinical data on 20 patients with advanced NSCLC treated with pembrolizumab immunotherapy, in which five hyperprogressing patients were identified, and NGS revealed CDKN2A/B deletion in 4/5 hyperprogressing patients.

### Immunohistochemistry staining of bladder cancer microarray

CD8 and PD-L1 staining was conducted using the BLCA tumor tissue microarray (HBlaU050CS01), which included 40 bladder cancer tissue samples and 10 adjacent peritumoral tissues, offered by Shanghai Outdo Biotech Company located in Shanghai, China. The positive ratio of CD8^+^ T cells were defined based on a comparison of the infiltration ratio of CD8^+^ cells within each nest to the number of total cells within each nest. Only the proportion of strongly positive cells were recorded, while the proportion of weakly positive cells was disregarded. The ethics committee of Shanghai Outdo Biotech Company granted approval for this study. Immunohistochemistry of the tumor tissue microarray was performed by Biossci Company in Hubei, China. An antibody against IL4I1 (ab222102) was purchased from Abcam company. For IL4I1 and PD-L1, we performed a semi-quantitative evaluation for staining intensity, categorizing it as negative (0), weakly positive (1^+^), moderately positive (2^+^), or strongly positive (3^+^), and determining the percentage of positive cells present. To determine the histochemistry score (H-score) for each observed tissue component (cytoplasm and nucleus), we multiplied the intensity score (ranging from 0 to 3) and the percentage of positive cells (ranging from 0 to 100).

### Anticipation of the BLCA molecular subtypes

To analyze the molecular subtypes in the samples and establish the correlation between IL4I1 expression and molecular subtypes, we utilized a range of subtype systems such as CIT, Lund, MDA, TCGA, Baylor, UNC, and Consensus subtypes and determined each sample’s subtype using consensusMIBC and BLCAsubtyping R packages (Damrauer et al., 2014; Rebouissou et al., 2014; Robertson et al., 2017; Marzouka et al., 2018; Mo et al., 2018; Kamoun et al., 2020). The molecular subtype of BLCA can be classified into binary subtype, including basal subtype and luminal subtype (Kamoun et al., 2020). The Basal subtype, which is considered more aggressive, also shows an increased response to certain treatments such as immunotherapy and anti-EGFR therapy. We also have gathered a total of 12 specific bladder cancer gene signatures (Kamoun et al., 2020). To assess the predictive precision of IL4I1 in identifying BLCA molecular subtypes, we created receiver operating characteristic (ROC) curves.

### Evaluating the correlation between IL4I1 and different treatment effectiveness

A set of gene signatures positively correlated with clinical response to anti-PD-L1 (atezolizumab) in BLCA from Mariathasan’s study (Mariathasan et al., 2018). Significantly, the determining factors for neoadjuvant chemotherapy in BLCA encompass the genetic mutation status of various pivotal genes like RB1, ATM, ERBB2, ERCC2, and FANCC (van Allen et al., 2014; Groenendijk et al., 2016; Pietzak et al., 2019; Singla et al., 2019). Additionally, we gathered additional therapeutic signatures, such as oncogenic pathways that could contribute to a non-inflamed TME, the EGFR ligands and radiotherapy predicted pathways. We used the ‘GSVA’ R package to calculate the enrichment scores for these signatures (Hänzelmann et al., 2013). Lastly, we acquired target genes of drugs employed in various therapeutic approaches by Drugbank (Wishart et al., 2018) database (https://www.drugbank.com/) and examined their association with IL4I1.

### Statistical analysis

To evaluate differences among continuous variables, either the Wilcoxon ranking test or Kruskal Wallis test was utilized, depending on the quantity of groups, whereas the chi-square test was employed to examine distinctions among categorical variables. The Pearson correlation analysis was utilized to quantify correlation. The survival cruve were generated using the Kaplan-Meier method and the prognostic value were obtained via univariate Cox regression. Statistical analyses were all conducted using R project (version 4.2.3). A significance level of 0.05 was used, and *p*-values less than this threshold were considered statistically significant.

## Results

### Identifying differential expressed genes (DEGs) for inflamed and non-inflamed phenotypes

**FIGURE 2:**
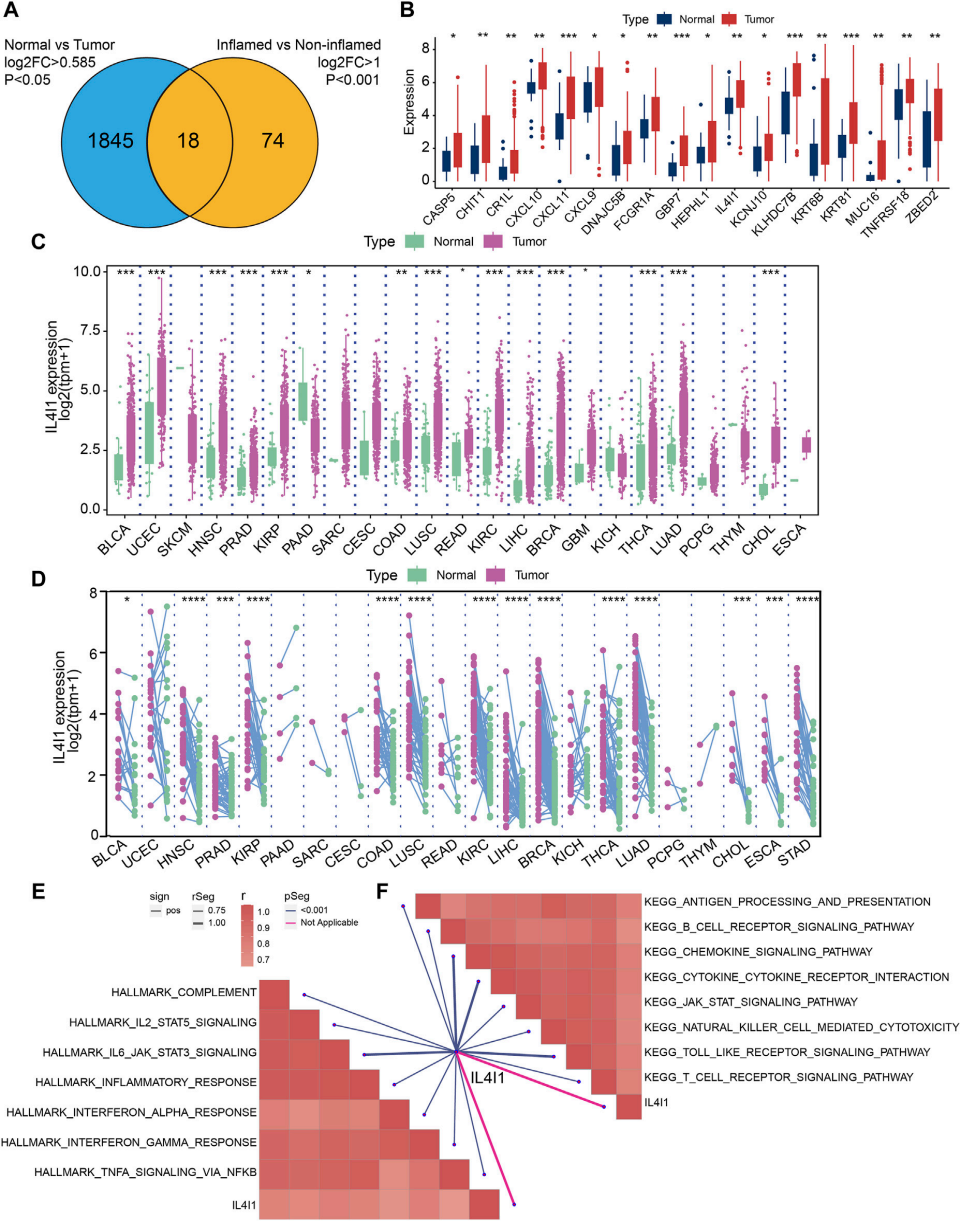
Identifying differential expressed genes (DEGs) for inflamed and non-inflamed phenotypes. **(A)** Common upregulated DEGs of TCGA-BLCA cohort and IMvigor210 cohort. **(B)** The expression of 18 DEGs in normal and tumor tissues of BLCA. **(C,D)** Pan-cancer expression pattern of IL4I1. GSEA for IL4I1 in Hallmark gene sets **(E)** or in KEGG gene sets **(F)** based on the TCGA-BLCA cohort.

Firstly, 1863 differential expressed genes between normal and tumor samples in the TCGA cohort were identified, followed by 92 DEGs between inflamed tumor samples and non-inflamed tumor samples in the IMvigor210 cohort. After crossing the DEGs from the two cohorts, we finally identified 18 common DEGs (Figure 2A). Figure 2B demonstrated the relative expression of these 18 DEGs. For these genes, IL4I1, CXCL9, CXCL10, CXCL11, KLHDC7B, and TNFRSF18 had a relative high expression in tumor samples. Here, we focused on the role of IL4I1 expression in bladder cancer. Then, we explored the pan-cancer expression pattern of IL4I1 by analyzing the different expression between normal and tumor tissues in 23 tumor types. It was found that the expression of IL4I1 was significantly higher in tumor tissues than normal tissues in most tumor types such as BLCA, HNSC, PRAD, KIRP, and so on (Figure 2C). The paired analysis showed the same trend (Figure 2D). Subsequently, we performed GSEA for IL4I1 in terms of Hallmark gene sets or KEGG gene sets based on the TCGA-BLCA cohort. As demonstrated, IL4I1 was significantly positively correlated with immune-related pathways, including IL-2-STAT5 signaling, IL4-JAK-STAT3 signaling and antigen processing and presentation (Figures 2E, F).

### Pan-cancer expression patterning and immunological function of IL4I1

**FIGURE 3:**
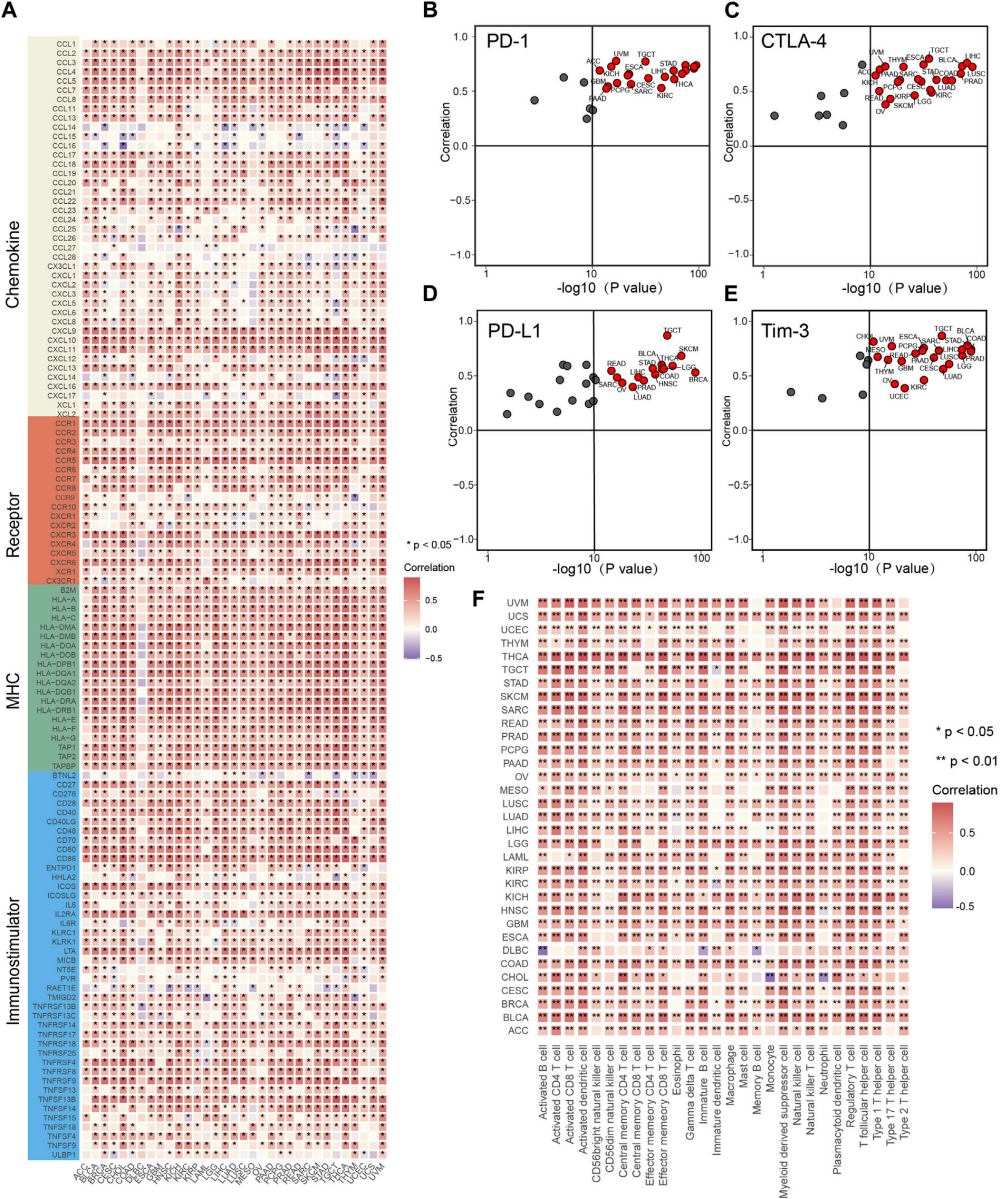
The effect of IL4I1 on immunological status in pan-cancers. **(A)** The heat map shows the relationship between IL4I1 and 121 immunomodulators (chemokines, receptors, MHC and immunomodulators). **(B–E)** The relationship between IL4I1 and four immune checkpoints, PD-1, CTLA-4, PD-L1, and Tim-3. These dots represent types of cancer. The vertical axis means the Pearson correlation, while the horizontal axis means -log10 (*p*-value). **(F)** The relationship between IL4I1 and 28 tumor-associated immune cells calculated by the ssGSEA algorithm. The color represents the correlation coefficient. (*p < 0.05; **p < 0.01).

We have demonstrated that IL4I1 is highly expressed in a variety of tumor tissues, prompting us to explore the prognostic value of IL4I1. We assessed the prognostic value of IL4I1 through univariate cox analysis and survival curves. Although IL4I1 has no prognostic value in bladder cancer, its overexpression was observed as a significant risk factor for ACC, GBM, KIRC, KIRP, LAML, LGG, LIHC, THYM, and UVM in terms of overall survival (OS), while a protective effect was observed only in SKCM (Supplementary Figure S1). The analysis of progression-free survival (PFS) revealed that high expression of IL4I1 was also found to be a contributing factor for GBM, KIRCK, IRPK, and LGG. In addition to SKCM, IL4I1 overexpression also had a significant protective effect on CESC, CHOL, and HNSC (Supplementary Figure S2). However, in the disease-free survival (DFS) analysis, only KIRP and THCA had a significant risk effect and were protective factors for OV (Supplementary Figure S3). Considering the results of GSEA suggests that IL4I1 is closely related to immune-related pathways, a comprehensive analysis was performed to identify the correlation between IL4I1 and immunological characteristics across various types of tumors. It was found that the expression of IL4I1 showed positive correlation with the vast majority of immunomodulators in almost all tumors, especially BLCA, HNSC, and KICH (Figure 3A). However, in certain tumors, such as THCT and UCEC, it is negatively correlated with some immunomodulators. In multiple types of cancers, we discovered a positive correlation between IL4I1 expression and various immune checkpoints, including PD-1, CTLA-4, PD-L1, and Tim-3 (Figures 3B–E). Next, the ssGSEA algorithm was utilized to analyze the infiltration level of TIICs. In contrast, except for DLBC and CHOL, IL4I1 showed a positive association with various types of TIICs in different tumors (Figure 3F). These findings suggested that the expression of IL4I1 holds immunological relevance and prognostic value across various types of tumors, suggesting that IL4I1 can serve as a target for immunotherapy.

### IL4I1 suggests an inflamed TME in BLCA

**FIGURE 4:**
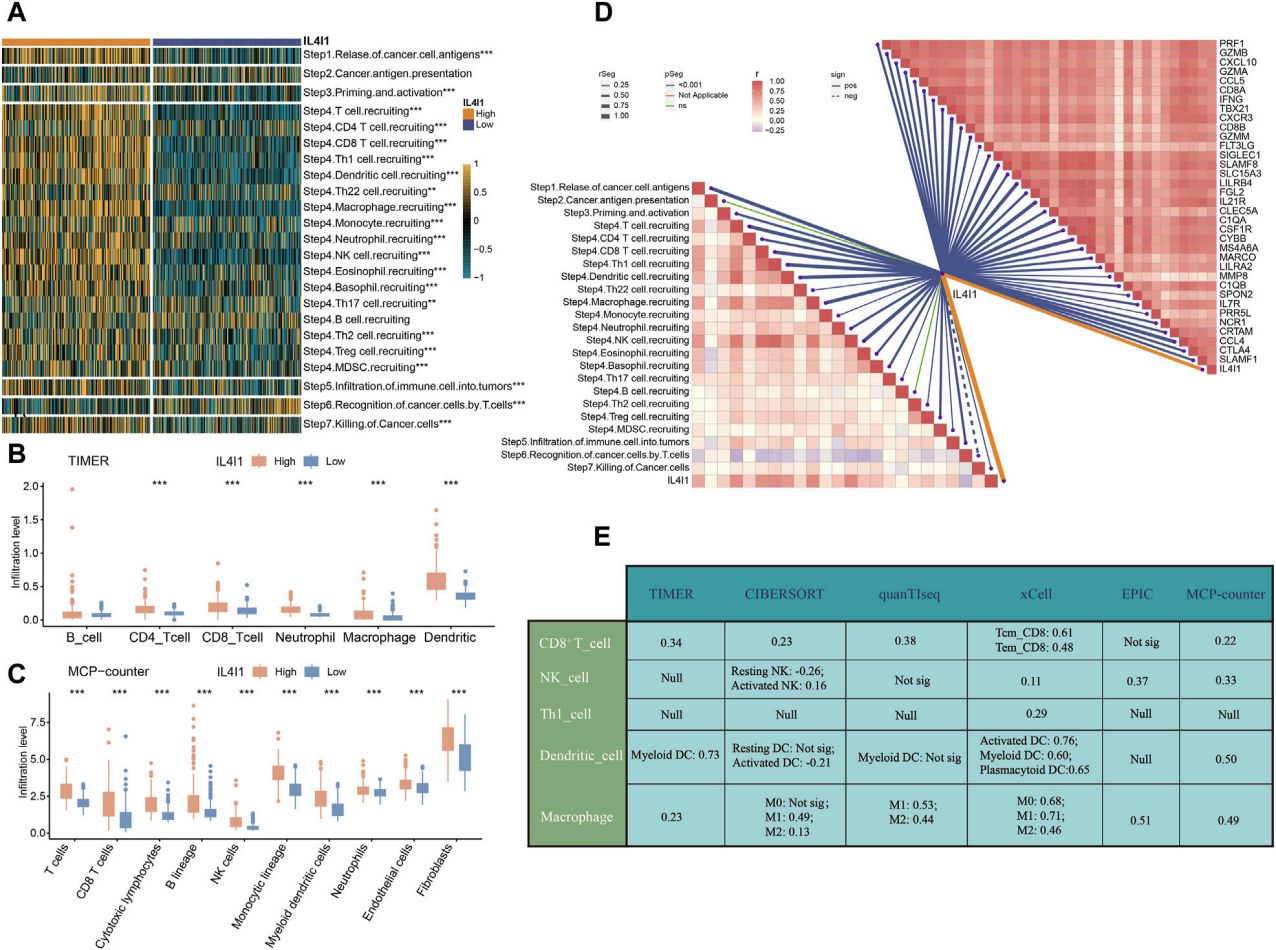
IL4I1 develops an inflamed TME in bladder cancer. **(A)** Correlation between IL4I1 and the activities of cancer immunity cycle. **(B)** Differences in the infiltration levels of five types of TIICs in the high- and low-IL4I1 groups using TIMER algorithm in bladder cancer. **(C)** Differences in the infiltration levels of five types of TIICs in the high- and low-IL4I1 groups using MCP-counter algorithm in bladder cancer. **(D)** Correlation between IL4I1 and the activities of cancer immunity cycle, effector genes of Tumor-associated immune cells mentioned above. **(E)** Correlation between IL4I1 and five types of TIICs using different algorithms. (*p < 0.05, **p < 0.01, ***p < 0.001, Not sig refers to not significant, Null means no data for analysis, T cm refers to central memory T cell, and T em refers to effector memory T cell).

Subsequently, we proceeded to analyze the immunological properties of IL4I1 in BLCA. Given that the immune system is a complex and interconnected network involving numerous molecules and pathways. To further evaluate the immunological functions of IL4I1, we assessed its impact on the cancer immune cycle. Our findings indicated that the enrichment score of most steps in the cancer immune cycle is upregulated in the high IL4I1 group (Figure 4A). The process involved release of cancer cell antigens (step 1), priming and activation (step 3), as well as trafficking of T cells to tumors (step 4) (except for B cell recruitment, all other steps including CD8^+^ T cell, DC, macrophage, Th1 cell, NK cell, MDSC, and Th17 cell recruitment showed increased activity). The connection between these processes and the infiltration of immune cells in the TME is evident through the heightened T cell infiltration into tumors (step 5) observed in the high-IL4I1 group. Furthermore, there was an upregulation in the activity of eliminating cancer cells (step 7) within the high-IL4I1 group. Significantly, the T cells' ability to identify cancer cells (step 6) was observed to be reduced in the high IL4I1 group, possibly because of the increased inhibitory immune checkpoints expression in this specific group.

Furthermore, we employed the TIMER and MCP-counter algorithms to quantify the infiltration levels of immune cells and analyzed their relationship with IL4I1. The results displayed a favorable association between IL4I1 manifestation and the levels of infiltration by CD8^+^T cells, macrophages, and dendritic cells (Figures 4B, C). Additionally, the expression of IL4I1 exhibited a positive relationship with the effector genes of TIICs (Figure 4D). Furthermore, we utilized six different algorithms to further investigate the relationship between IL4I1 and TIICs (including CD8^+^ T cells, NK cells, Th1 cells, dendritic cells, and macrophages) (Figure 4E).

To summarize, these findings indicated a notable correlation between the excessive expression of IL4I1 and the inflamed TME within BLCA.

### IL4I1 predicts clinical response and hyperprogression of ICB in BLCA

**FIGURE 5:**
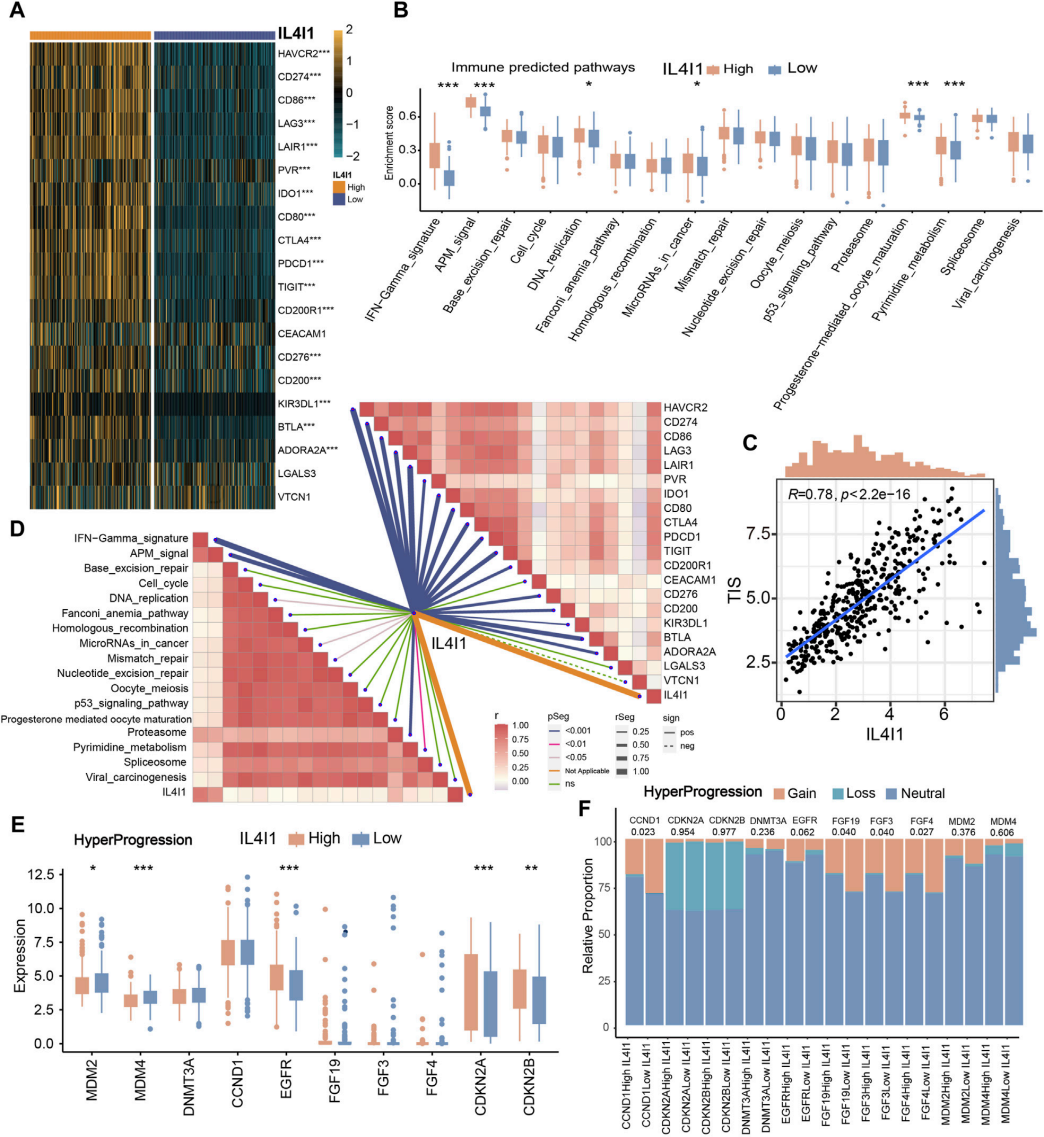
The role of IL4I1 in predicting the response to immunotherapy. **(A)** Correlation between IL4I1 and 20 immune checkpoints. **(B)** Differences in the enrichment scores of immunotherapy-predicted pathways in the high- and low-IL4I1 groups. **(C)** Correlation between IL4I1 and pan-cancer T cell inflamed score. **(D)** Correlation between IL4I1 and immunotherapy-predicted pathways, 20 immune checkpoints. **(E,F)** Differences in the expression and mutation status of hyperprogression-related biomarker in the high- and low-IL4I1 groups. (*p < 0.05, **p < 0.01, ***p < 0.001, ns means not significant, pos means positive correlation, and neg mean negative correlation).

Based on the previous description, we proposed that IL4I1 suggested an inflamed TME, thereby suggesting that high expression of IL4I1 may confer increased sensitivity to ICB therapy. Consequently, we embarked on an analysis to explore the association between IL4I1 and 20 inhibitory immune checkpoints. With the exception of LGALS3 and VTCN1, the expression levels of immune checkpoints exhibited significant upregulation. Correlation analysis further indicated a positive association between IL4I1 expression and these immune checkpoints (Figures 5A, D). Furthermore, IL4I1 exhibited positive correlations with certain pathway enrichment scores that are known to be favorable for immunotherapy (Figure 5B). Furthermore, there is a significant and positive association between IL4I1 and TIS (Figure 5C). The above results suggested that IL4I1 has the capacity to serve as a promising indicator for the effectiveness of ICB. Conversely, the high-IL4I1 group exhibited a lower incidence of hyperprogression. The correlation between IL4I1 and hyperprogression related genes was examined, and our results showed a notable decrease in the expression of genes that exhibited a positive correlation with the occurrence of hyperprogression in the high IL4I1 group. This reduction was observed in genes such as MDM2, MDM4, as well as a significant decrease in the rate of copy number amplification, including CCND1, FGF19, FGF3, and FGF4 (Figures 5E, F). Conversely, two predictors that exhibited a negative correlation with hyperprogression, namely CDKN2A and CDKN2B, exhibited significantly higher expression levels in the high-IL4I1 group compared to the low-IL4I1 group, along with a higher copy number amplification rate. These findings suggested that patients exhibiting high IL4I1 expression are less prone to experiencing hyperprogression associated with ICB treatment, thus making them more appropriate candidates for ICB therapy.

### IL4I1 expression could precisely predict the molecular subtypes and possible therapeutic strategies in BLCA

**FIGURE 6:**
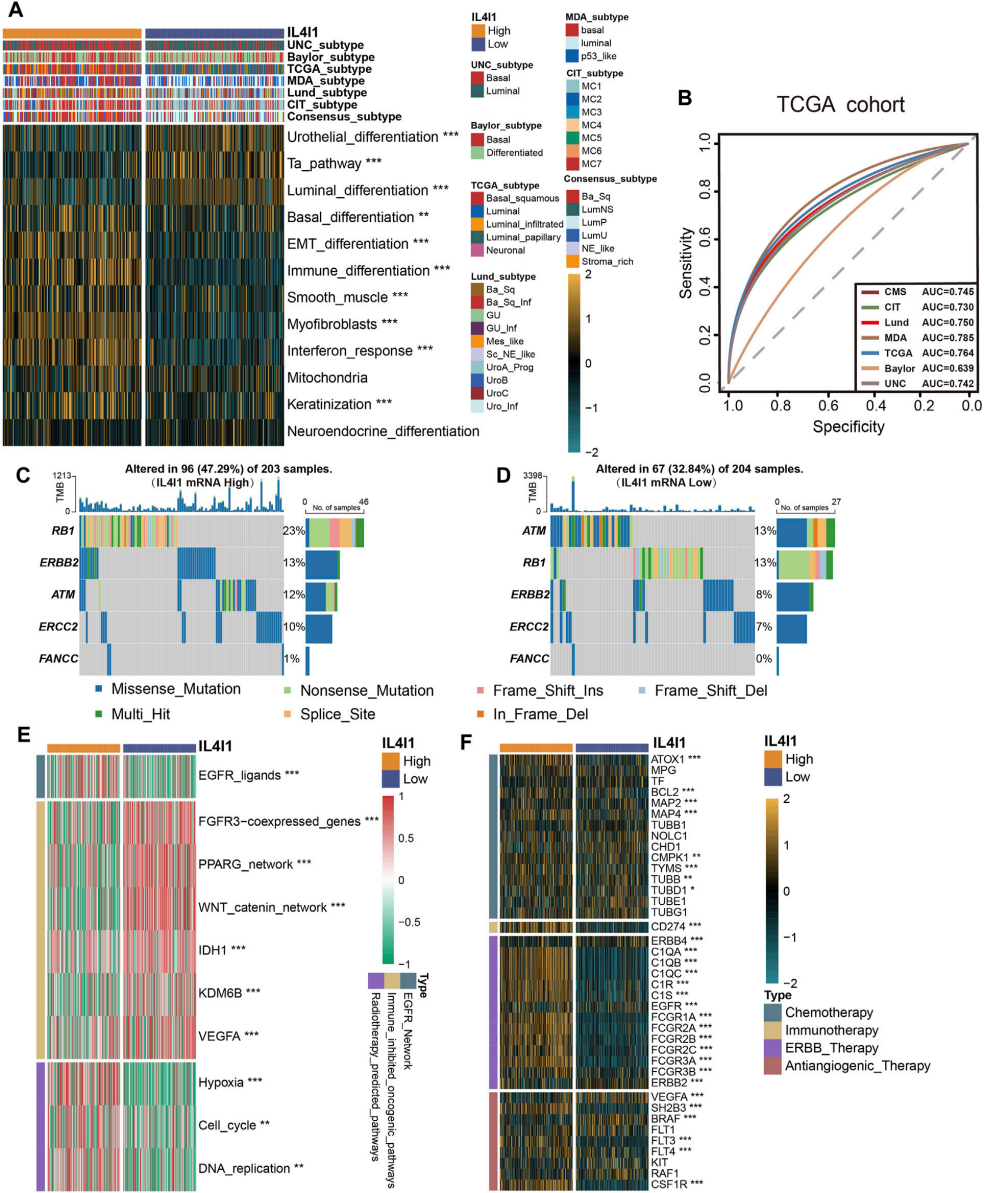
L4I1 can predict the molecular subtype and the curative effect of several treatments in bladder cancer. **(A)** Differences in the seven different subtype systems and bladder cancer signatures between high- and low- IL4I1 groups. **(B)** ROC curves measuring the predictive value about molecular subtypes using seven algorithms. **(C,D)** Differences in mutations of neoadjuvant chemotherapy-related genes in high- and low- IL4I1 groups. **(E)** Correlation between IL4I1 and the enrichment scores of several therapeutic-related signaling pathways such as targeted therapy. **(F)** Correlation between IL4I1 and drug-target genes of different therapy extracted from the Drugbank database. (*p < 0.05, **p < 0.01, ***p < 0.001).

The different molecular subtypes of BLCA affect the prognosis and treatment effect of patients. Hence, an analysis was conducted to examine the correlation between the IL4I1 expression and molecular subtypes across various classification systems. Figure 6A demonstrated that the high-IL4I1 group displayed a stronger preference for the basal subtype of BLCA in the findings. Furthermore, the low-IL4I1 group exhibited elevated enrichment scores for urothelial differentiation, the Ta pathway, and luminal differentiation (Figure 6A). The high-IL4I1 group exhibited elevated enrichment scores in basal differentiation, EMT differentiation, immune differentiation, smooth muscle, myofibroblast, interferon response, and keratinization. Moreover, apart from the Baylor system, all molecular subtypes exhibited an AUC value exceeding 0.7 (Figure 6B), signifying that IL4I1 has the ability to accurately anticipate the molecular subtype of BLCA, thus providing precise treatment guidance.

In addition to immunotherapy, IL4I1 can predict the efficacy of other therapeutic strategies, like neoadjuvant chemotherapy, radiotherapy, and various targeted therapies. We collected mutation data for molecules associated with neoadjuvant chemotherapy, various therapeutic signatures, and gene expression related to targeted drugs. In the high-IL4I1 group, the mutation frequency of specific genes (RB1, ERBB2, ERCC2) linked to neoadjuvant chemotherapy exhibited a higher rate (Figures 6C, D). Furthermore, the group with high IL4I1 levels demonstrated increased enrichment scores for pathways linked to EGFR ligands and radiotherapy. On the contrary, the group exhibiting low IL4I1 levels demonstrated an enrichment of oncogenic pathways, as depicted in Figure 6E. The activation of these pathways was found to be connected with a non-inflamed TME and resistance to immunotherapy (Peng et al., 2015; Sweis et al., 2016; Spranger and Gajewski, 2018). By utilizing the Drugbank database, we discovered an observation indicating significantly higher expression levels of numerous drug target genes in the high-IL4I1 group (Figure 6F). In particular, the high IL4I1 group exhibited a significant increase in nearly all aspects related to chemotherapy, immunotherapy, and ERBB target gene expression. And some target genes of anti-angiogenesis (including SH2B3, FTL3, FTL4, and CDF1R) were also significantly increased, while the expression levels of BRAF and RAF1 were low.

In general, these results suggested that BLCA patients with high IL4I1 expression may benefit more from neoadjuvant and adjuvant chemotherapy, radiotherapy, immunotherapy, and ERBB therapy.

### Validating the role of IL4I1 in GSE48075 and E-MTAB-4321 cohort

**FIGURE 7:**
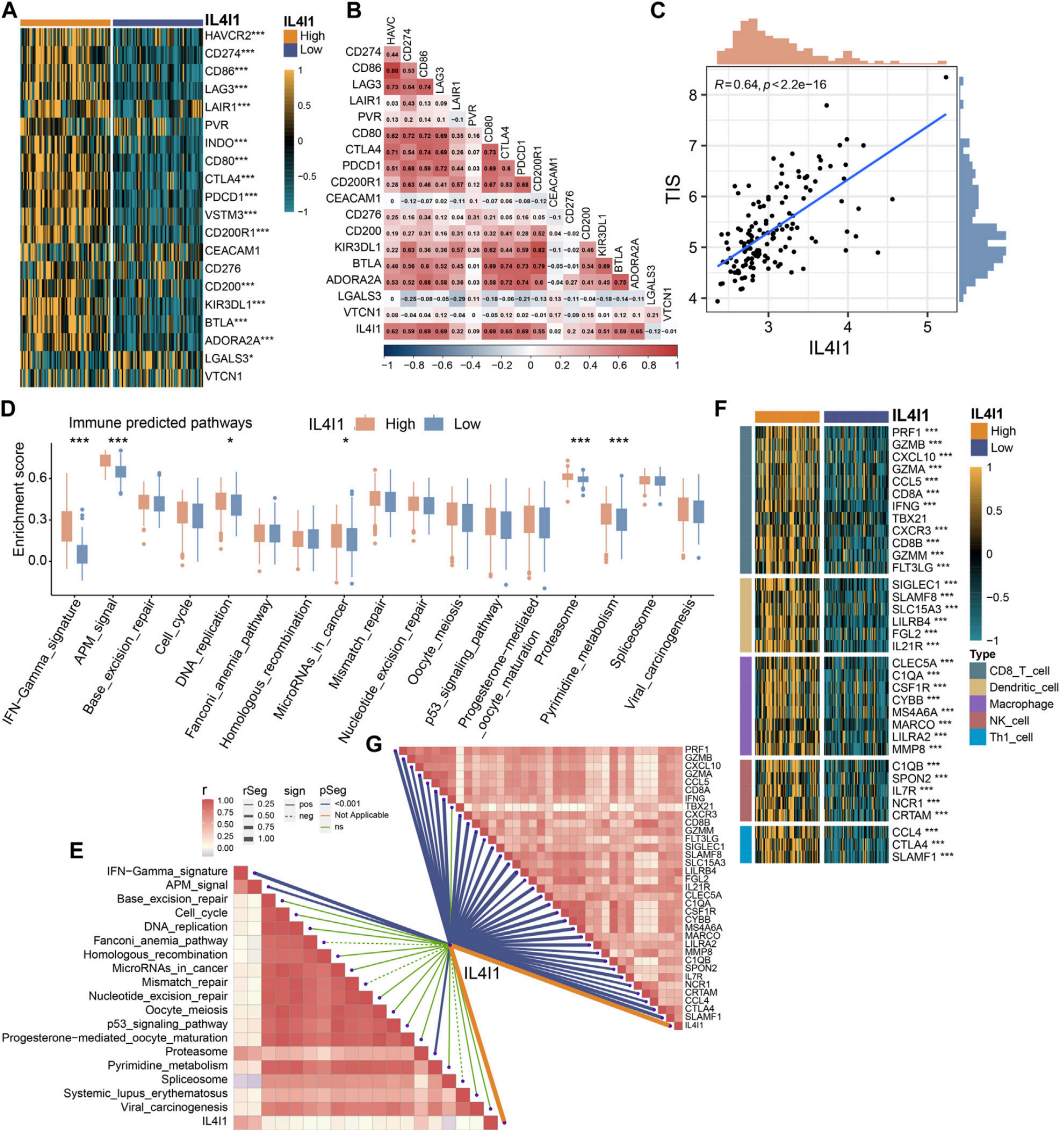
Validating the predictive ability of IL4I1 expression for immune therapy response in the GSE48075 cohort. **(A)** Differences in the expression of immune checkpoint in the high- and low-IL4I1 groups. **(B)** The correlation between IL4I1 expression and immune checkpoints by Pearson’s correlation analysis. **(C)** Correlation between IL4I1 expression and T cell-inflamed scores in the GSE48075 cohort. **(D)** Differences in the enrichment scores of immune predicted pathways between the high- and low-IL4I1 groups. **(E)** The correlation analysis between IL4I1 expression and enrichment scores of immune predicted pathways. **(F)** Comparison for expression of effector genes in the high- and low-IL4I1-expression groups. **(G)** The correlation analysis between IL4I1 expression and expression of effector genes (*p < 0.05, **p < 0.01, ***p < 0.001, ns means not significant, pos means positive correlation, and neg mean negative correlation).

**FIGURE 8:**
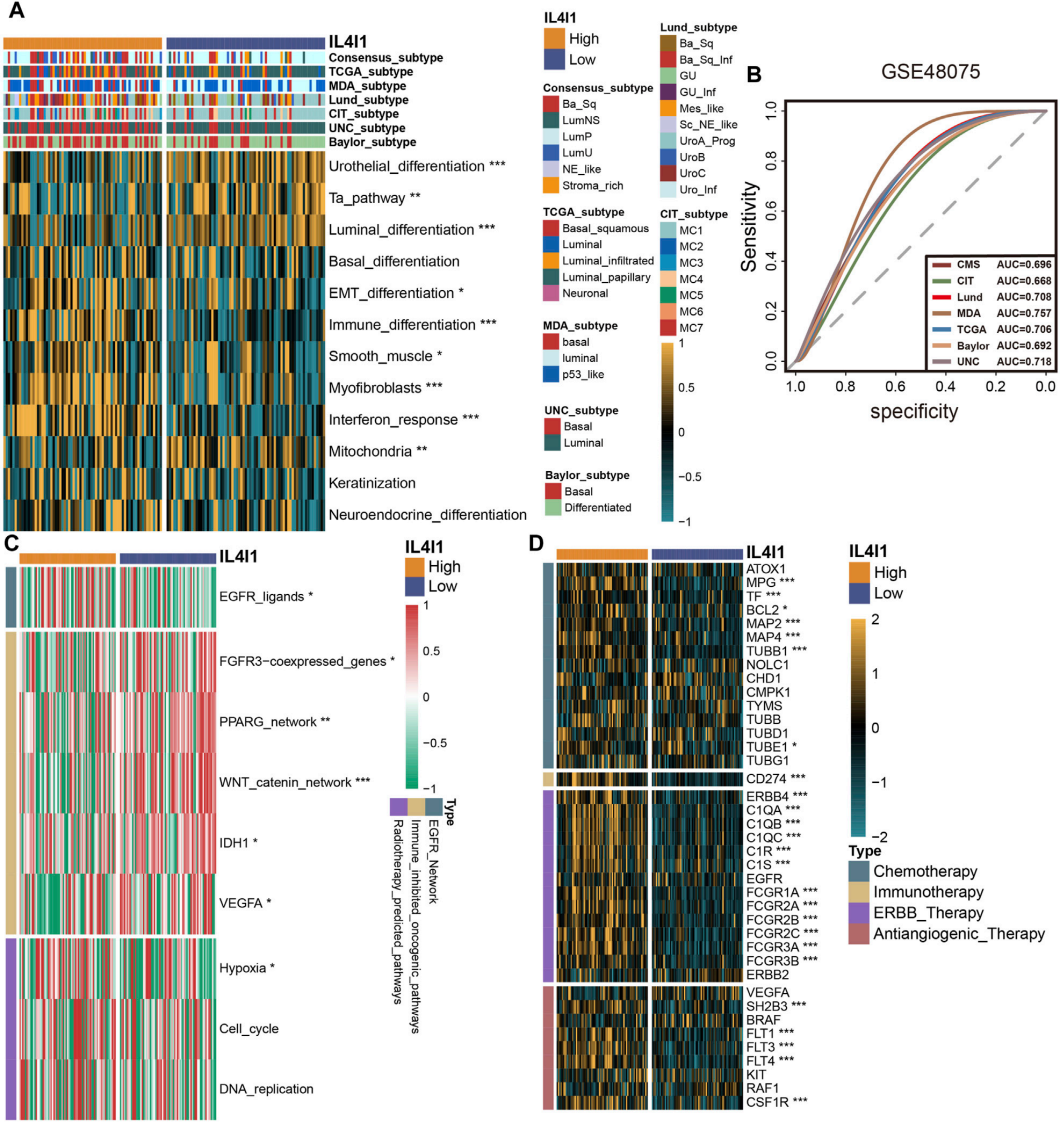
Validating the predictive ability of IL4I1 expression for the molecular subtype and response for various treatment strategies of bladder cancer in the GSE48075 cohort. **(A)** Difference of bladder cancer molecular subtypes in patients with different IL4I1 expression levels calculated via multiple algorithms. **(B)** Receiver operating characteristics (ROC) curve and area under curve (AUC) for IL4I1 in molecular subtype prediction in the GSE48075 cohort. **(C)** Difference for enrichment scores of various therapeutic signatures in the high- and low-IL4I1 groups. **(D)** Difference for expression of various drug-targeted genes in the high- and low-IL4I1 groups. (*p < 0.05, **p < 0.01, ***p < 0.001).

To provide more robust results, we performed a validation analysis in the GSE48075 cohort and E-MTAB-4321 cohort. There was a significant increase in the expression of the majority of immune checkpoints in patients with high IL4I1 expression levels (Figure 7A; Supplementary Figure S4A), and correlation analysis also showed a positive correlation (Figure 7B). Similarly, the expression of IL4I1 was positively correlated with TIS (Figure 7C). Several immunotherapy positive predictable pathways also significantly enriched in high-IL4I1 group (Figure 7D, Supplementary Figure S4B) and are positively associated with IL4I1 (Figure 7E). Furthermore, the majority of effector genes in TIICs exhibited a positive correlation with IL4I1, as depicted in Figures 7F, G; Supplementary Figure S4C. Two external cohort revealed a significant correlation between elevated IL4I1 expression and the basal molecular subtypes of BLCA, thereby validating IL4I1’s potential as a predictive indicator for BLCA molecular subtypes (Figures 8A, B; Supplementary Figures S4D, E). For several therapeutic strategies, IL4I1 also showed its predictive value. The high-IL4I1 group showed significant enrichment of EGFR and radiotherapy predictive pathways (Figure 8C; Supplementary Figure S4F), and IL4I1 was positively correlated with the expression of drug target for chemotherapy, immunotherapy, ERBB and anti-angiogenic therapy (Figure 8D). The validation of the GSE48075 and E-MTAB-4321 cohort further confirmed IL4I1 suggested an inflamed TME and the potential of IL4I1 to predict molecular subtypes and several therapeutic strategies for BLCA.

### Validating of IL4I1′role from immunohistochemistry and the IMvigor210 cohort

**FIGURE 9:**
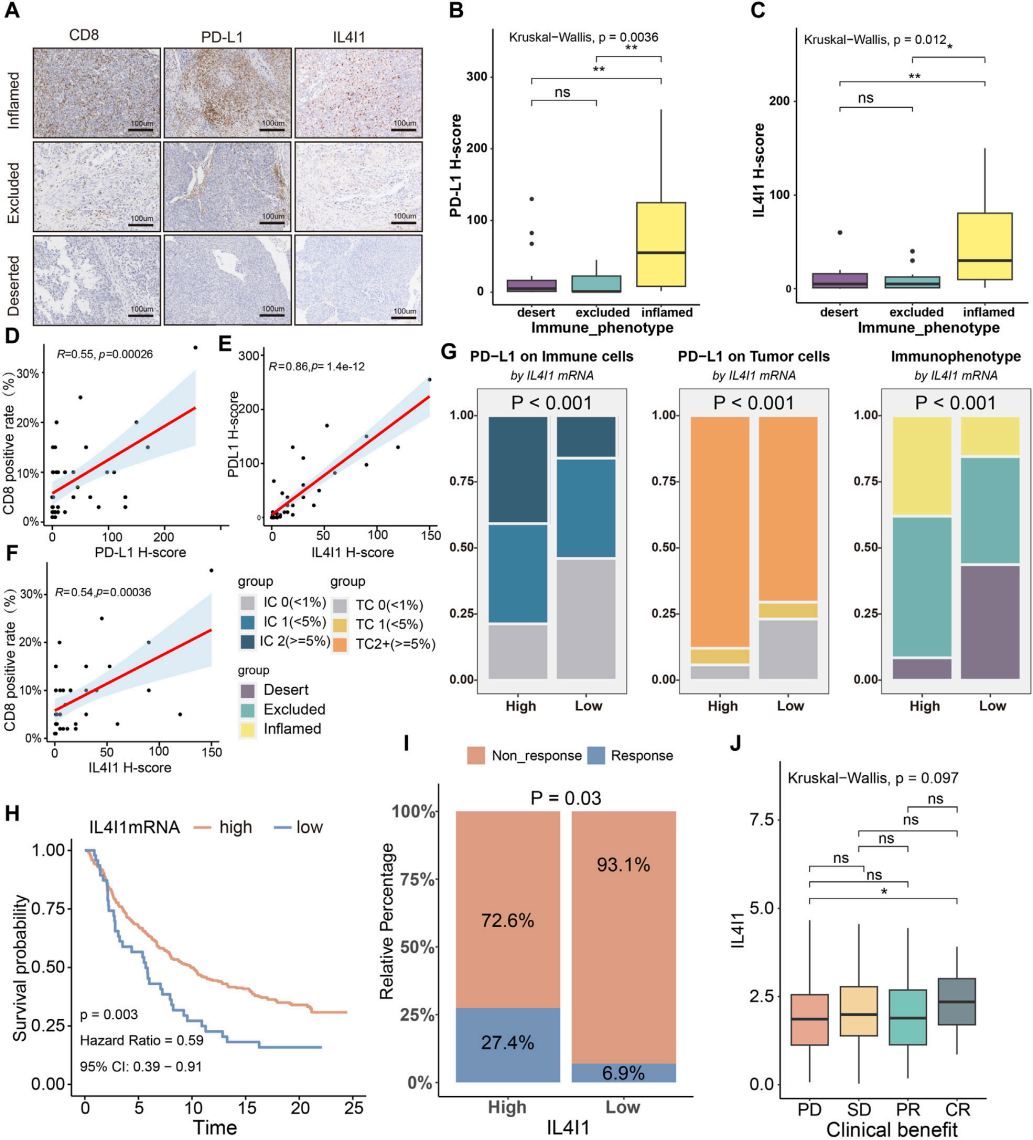
Correlation between IL4I1, the immune phenotype and the clinical response of immunotherapy in BLCA. **(A)** Expression of IL4I1, CD8, and PD-L1 in the BLCA TMA cohort was measured using immunohistochemistry. Representative images of CD8, PD-L1, and IL4I1 in three immune phenotypes were displayed. The scale bars correspond to 200 μm. **(B,C)** PD-L1 H score and IL4I1 positive rate (detected using immunohistochemistry) in the three phenotypes of the TMA cohort. **(D,E)** Correlation between PD-L1 H score, IL4I1 positive rate and CD8 positive rate. **(F)** Correlation between IL4I1 positive rate and PD-L1 H score. **(G)** Differences in the expression of PD-L1 on the immune cells and tumor cells, and immunophenotype in the high- and low-IL4I1 groups. **(H)** Kaplan-Meier analysis for overall survival of bladder cancer patients with ICB therapy based on IL4I1. **(I)** Differences in the response to immunotherapy in the high- and low-IL4I1 groups. **(J)** Correlation between Siglec15 and the clinical response of cancer immunotherapy in the IMvigor210 cohort. (*p < 0.05, **p < 0.01, ns refers to not significant, TC refers to tumor cells, IC refers to immune cells; PD: Progressed disease; SD: Stable disease; PR: Partial response; CR: Complete response).

Next, we performed immunohistochemical staining of BLCA and classified the tumor tissue microarray cohort into inflamed, excluded, and desert subtypes (Ott et al., 2019) derived from the spatial distribution pattern of CD8^+^ T cells (Figure 9A). In the inflamed subtype, the H-score of IL4I1 and PD-L1 were found to be higher comparison to the other two subtypes (Figures 9B, C). Figures 9D–F showed that there was a positive association between the H-score of PD-L1 and the rate of CD8 positivity, while the H-score of IL4I1 was positively linked to the H-score of PD-L1 and the positive rate of CD8. In the IMvigor210 cohort (a BLCA immunotherapy cohort), according to the clinical efficacy of the patients, the cohort was divided into PR/CR group and PD/SD group, and the correlation of IL4I1 with immunological characteristics and its predictive value for molecular subtypes and therapy were explored respectively. And we also arrived a similar conclusion. (Supplementary Figures S6, S7). Additionally, we conducted investigations into the involvement of IL4I1 in immunotherapy. As anticipated, the high-IL4I1 group displayed notably elevated percentages of IC2 (immune cells with the utmost PD-L1 level) and TC2 (tumor cells with the utmost PD-L1 level) (Figure 9G), aligning with our expectations for patients who received anti-PD-1 therapy. In addition, the high-IL4I1 group had a higher survival rate and response rate to anti-PD-L1 therapy, and IL4I1 expression was higher in the complete response group (Figures 9H–J). These findings collectively suggested that IL4I1 was closely related to the formation of inflamed TME and has a certain value in predicting the response to immunotherapy in bladder cancer. Additionally, we collected six immunotherapy cohorts, including five melanoma ICB treatment cohorts and one adoptive T cell therapy cohort to investigate the predictive role of IL4I1 in immunotherapy response. Although not reaching statistical significance, it is noteworthy that in cohorts such as Gide 2019, Nathanson2017 post, and GSE78220, the high IL4I1 group exhibited higher response rates and thus a more favorable prognosis, consistent with our findings in bladder cancer. In contrast, in cohort Nathanson2017 pre, the high IL4I1 group displayed a lower response rate compared to the low IL4I1 group, resulting in a poorer prognosis (Supplementary Figures S8A–F).

## Discussion

Secreted L-amino acid oxidase IL4I1 is primarily produced by inflammatory antigen presenting cells, such as monocytes, macrophages, and dendritic cells (Marquet et al., 2010; Park et al., 2017). By decomposing phenylalanine, IL4I1 can change the biochemical environment of immune cells (Molinier-Frenkel et al., 2019), thereby regulating the immune response. According to recent research, IL4I1 has the ability to control the immune response by impacting the growth of immune cells (Chavan et al., 2002; Boulland et al., 2007; Houston et al., 2015; Pluchino and Peruzzotti-Jametti, 2016; Romagnani, 2016; Bod et al., 2018; Sadik et al., 2020; Castellano et al., 2021). In this study, we elucidated the expression pattern of IL4I1 in pan cancer and its association with poor prognosis in various tumors. Furthermore, we observed a positive correlation between IL4I1 and immunological characteristics in BLCA, suggesting that IL4I1 may serve as a representative marker for an inflamed tumor microenvironment. Building upon this foundation, we further demonstrated that IL4I1 can accurately predict immunotherapy response and BLCA molecular subtypes.

In this study, we revealed that IL4I1 was a prognostic factor in various cancer types. For instance, the survival curve indicates that patients with high IL4I1 expression obtained an unfavorable prognosis in LGG and GBM which was in consist with research of Feng Ye et al. IL4I1 was predominantly expressed on tumor associated macrophages (TAMs) in glioma. IL4I1-induced polarization of M2 macrophages can promote tumor invasion and metastasis (Ye et al., 2023). Similarly, IL4I1 was also found to be oncogenic effects in clear cell renal cell carcinoma through polarization of M2-like macrophages via JAK1/STAT3 signaling (Lin et al., 2023). Regretfully, the prognostic value of IL4I1 in bladder cancer was not observed in terms of both OS, PFS and DFS. Considering that IL4I1 is mainly expressed by TAMs, it is necessary to identify specific markers of IL4I1^+^ TAMs via scRNA-seq in the future, and further clarify whether it could serve as prognostic value in bladder cancer.

IL4I1 was positively correlated with the expression of multiple immunomodulators and immune checkpoints in most tumor types, thus validating its regulatory function in the immune system. Notably, immune cell infiltration analysis highlights a positive association between IL4I1 and immune cell infiltration across nearly all tumor types, particularly in BLCA, LGG, SKCM, THCA, and UVM. However, it’s worth noting that in SKCM, there are indications that IL4I1 inhibits CD8^+^ T cell infiltration, potentially mediating resistance to immunosuppressive agents (Bod et al., 2017). Given the complicated TME, the immunological characteristics of IL4I1 exhibit varying outcomes across distinct tumor types, and further validation through *in vitro* and *in vivo* experiments is warranted.

Specific chemokines play a dual role in modulating the migration of immune cells into the tumor microenvironment and directly targeting non-immune cells, such as tumor cells and vascular endothelial cells. For instance, CXC-chemokine ligand 9 (CXCL9) and CXCL10 bind to CXC-chemokine receptor 3 (CXCR3) expressed on effector CD8^+^ T cells, TH1 cells, and NK cells, facilitating their migration into the tumor site (Nagarsheth et al., 2017). Studies have demonstrated that the upregulation of CXCL9 and CXCL10 expression enhances CD8^+^ T cell infiltration in tumors, leading to improved prognosis in ovarian and colon cancers (Zhang et al., 2003; Pagès et al., 2005; Sato et al., 2005; Galon et al., 2006). Our study revealed a positive correlation between IL4I1 expression and various immunomodulators associated with T cell activation and expansion in bladder cancer, including CXCL9 and CXCL10. And the cancer immunity cycle encompasses seven steps ranging from release of cancer cells antigen to the killing of cancer cells. Disruption or diminished activity in any of these steps can impede the maximization of anti-cancer immune responses. For instance, impaired recognition and presentation of tumor antigens, as well as ineffective recruitment and infiltration of tumor infiltrating immune cells into the tumor microenvironment, ultimately lead to the inadequate killing of cancer cells (Chen and Mellman, 2013; Motz and Coukos, 2013). In this study, we identified a significant positive correlation between IL4I1 expression and several steps of the cancer immunity cycle, including T-cell recruitment and immune cell infiltration into tumor tissues. The inflamed TME in bladder cancer is characterized by the presence of CD4^+^ and CD8^+^ T cells localized in proximity to tumor cells (Spranger et al., 2013; Gajewski, 2015; Fehrenbacher et al., 2016). Since IL4I1 is positively correlated with the expression of immunomodulators and the activity of most steps of the cancer immunity cycle was significantly upregulated in the high IL4I1 group, these implied that IL4I1 is strongly associated with the inflamed tumor microenvironment. Consequently, T cells, NK cells, and macrophages, collectively termed tumor infiltrating immune cells (TIICs), exhibited increased infiltration in the high IL4I1 expression group, a phenomenon validated through immunohistochemical. Moreover, the impact of IL4I1 on the TME appears to vary across different tumor types. For instance, in melanoma, IL4I1 overexpression was associated with a reduction in CD8 mRNA levels and CD8^+^ T cell infiltration (Bod et al., 2017). Tong Li et al. discovered that inhibition of IL4I1 via Thymol could enhance CD8^+^ T cell infiltration and reshape the inflamed TME. This approach had the potential to improve the efficacy of anti-PD-1 antibody treatment in lung adenocarcinoma (Li et al., 2020). This variability may be attributed to tumor heterogeneity and histological differences, which reminds us that immunological functions of IL4I1 in different tumors need to be further explored. In summary, our findings indicate that IL4I1 delineates an inflamed TME in bladder cancer, highlighting its potential as a key regulator of immune responses in the context of tumor progression.

Another feature of the inflamed TME is the elevated expression of suppressive immune checkpoints, which is induced by pre-existing tumor infiltrating immune cells (Spranger et al., 2013). Under physiological conditions, immune checkpoint molecules regulate the immune system by stimulating and suppressing the immune response; however, in tumors, their interaction suppresses pre-existing anticancer immunity, leading to immune escape. The clinical efficacy of immune checkpoint blockade (ICB) depends on the pre-existing anticancer immunity (Pardoll, 2012; Wykes and Lewin, 2018; Anandappa et al., 2020). Our results demonstrated that the high IL4I1 group exhibited upregulated expression of most immune checkpoints. This suggests that this group of patients has pre-existing anticancer immunity and is more likely to achieve better clinical efficacy of ICB. Additionally, the enrichment scores of immune predictive pathways were higher in the high IL4I1 group, and IL4I1 demonstrated a positive correlation with Tumor Inflammation Signature (TIS), which is used to predict ICB efficacy (St Paul and Ohashi, 2020). In the IMvigor210 cohort, the high IL4I1 group exhibited superior prognoses and higher response rates to immunotherapy. Collectively, these findings indicate that high IL4I1 expression may be more conducive to improved response to ICB in BLCA. Additionally, the correlation of IL4I1 with TME and immunotherapy varies across other tumor types. For instance, Tong Li et al. demonstrated that IL4I1 silencing in LUAD leads to increased infiltration of CD8^+^ T cells. Targeting IL4I1 with Thymol can reshape TME and enhance sensitivity to immunotherapy (Li et al., 2020). Hirose et al. found that IL4I1 overexpression defined an immunosuppressive TME in melanoma leading to resistance to anti-PD-L1 therapy (Hirose et al., 2024), which was not entirely consistent with the results of six melanoma immunotherapy cohorts in our study. It is worth mentioning that Matusiak’s study revealed the IL4I1^+^ TAM were involved in phagocytosis of tumor cells within the colon cancer TME and were associated with favorable clinical outcomes (Matusiak et al., 2024), which provided ideas for targeting specific TAM. Therefore, further investigation is required to explore the impact of IL4I1 on the inflamed TME in various types of tumors, as well as its potential as a predictive marker for the efficacy of immunotherapy.

We explored the association between IL4I1 and molecular subtypes of BLCA, considering that basal subtypes of BLCA are more prone to chemotherapy, ICB therapy, and EGFR-based targeted therapy. (Sjödahl et al., 2012; Choi et al., 2014; Damrauer et al., 2014; Rebouissou et al., 2014; Robertson et al., 2017; Mo et al., 2018; Kamoun et al., 2020). Our findings revealed that BLCA patients with elevated IL4I1 expression had a higher likelihood of displaying the basal subtype. Additionally, there was a notable enrichment of the associated pathways. The findings indicated that IL4I1 can be utilized for predicting molecular subtypes of BLCA, and the AUC value serves to validate the precision of the findings. On the other hand, an increased mutation rate of neoadjuvant chemotherapy-related genes and a greater enrichment score of EGFR ligand and chemotherapy predictive pathway were observed in association with elevated levels of IL4I1. And the expression of most drug target genes was higher in the high IL4I1 group according to results from the Drugbank database. Through these results, we believe that IL4I1 has predictive value in the selection of multiple therapeutic approaches targeting BLCA.

The study also has some limitations. Our results are mainly based on bioinformatics analysis of public databases. Although we integrate multiple data sources and different types of data for validation, the generation of data is usually based on statistical methods and models, and the interpretation of the results needs to consider the biological context and functional annotation. Nevertheless, there remain unexplored aspects of the functionalities of genes and pathways, presenting specific obstacles for data analysis that necessitate conducting experimental validation to elucidate the immunological function of IL4I1. Additionally, due to the absence of follow-up information and immunotherapy efficacy data for the patient sample from Shanghai Outdo Biotech Company, the effectiveness data for ICB treatment in the IMvigor210 cohort might be influenced by random effects and biases, and have not undergone independent validation.

In conclusion, this study shows that IL4I1 represents an inflamed TME in BLCA, and can also predict the molecular subtypes of BLCA and the clinical response to immunotherapy, providing guidance for clinical treatment options.

## Data Availability

The original contributions presented in the study are included in the article/Supplementary Materials, further inquiries can be directed to the corresponding author.

## Supplementary material

SUPPLEMENTARY FIGURE S1 The prognostic value of IL4I1 in pan-cancers in terms of overall survival (OS). **(A)** Forest map of univariate cox analysis for IL4I1 in cancers in terms of OS. **(B–I)** Kaplan-Meier survival curve for IL4I1 in various cancer types in terms of OS.

SUPPLEMENTARY FIGURE S2 The prognostic value of IL4I1 in pan-cancers in terms of progression-free survival (PFS). **(A)** Forest map of univariate cox analysis for IL4I1 in pan-cancers in terms of PFS. **(B–I)** Kaplan-Meier survival curve for IL4I1 in various cancer types in terms of PFS.

SUPPLEMENTARY FIGURE S3 The prognostic value of IL4I1 in pan-cancers in terms of Disease-free survival (DFS). **(A)** Forest map of univariate cox analysis for IL4I1 in pan-cancers in terms of DFS. **(B–I)** Kaplan-Meier survival curve for IL4I1 in various cancer types in terms of DFS.

SUPPLEMENTARY FIGURE S4 Validation in the E-MTAB-4321 cohort. **(A)** Correlations between IL4I1 and immune checkpoint. **(B)** Correlation between IL4I1 and immunotherapy predicted pathway. **(C)** Correlation between IL4I1 and effector genes associated with five types of TIIC. **(D)** Correlations between IL4I1 and molecular subtypes of BLCA. **(F)** Correlations between IL4I1 and several therapeutic signatures.

SUPPLEMENTARY FIGURE S5 Validation in IMvigor210 cohort. **(A)** Differences in the expression of immunomodulators between high and low IL4I1 groups. **(B)** Differences in the expression of effector genes associated with five types of TIIC between high and low IL4I1 groups. **(C)** Differences in the expression of immune checkpoint between high and low IL4I1 groups. **(D)** Correlation between IL4I1 and immunotherapy predicted pathway. **(E)** Correlations between IL4I1 and molecular subtypes of BLCA. **(F)** ROC curve and AUC for IL4I1 in molecular subtype prediction in the IMvigor210 cohort. **(G)** Correlations between IL4I1 and several therapeutic signatures.

SUPPLEMENTARY FIGURE S6 Validation in PR/CR group of IMvigor210 cohort. **(A)** Differences in the expression of immunomodulators between high and low IL4I1 groups. **(B)** Differences in the expression of effector genes associated with five types of TIIC between high and low IL4I1 groups. **(C)** Differences in the expression of immune checkpoint between high and low IL4I1 groups. **(D)** Correlation between IL4I1 and immunotherapy predicted pathway. **(E)** Correlations between IL4I1 and molecular subtypes of BLCA. **(F)** Correlations between IL4I1 and several therapeutic signatures.

SUPPLEMENTARY FIGURE S7 Validation in PD/SD group of IMvigor210 cohort. **(A)** Differences in the expression of immunomodulators between high and low IL4I1 groups. **(B)** Differences in the expression of effector genes associated with five types of TIIC between high and low IL4I1 groups. **(C)** Differences in the expression of immune checkpoint between high and low IL4I1 groups. **(D)** Correlation between IL4I1 and immunotherapy predicted pathway. **(E)** Correlations between IL4I1 and molecular subtypes of BLCA. **(F)** Correlations between IL4I1 and several therapeutic signatures.

SUPPLEMENTARY FIGURE S8 IL4I1 predicted the response to immunotherapy in several immunotherapy cohorts. **(A-F)** Five ICB cohorts (VanAllen 2015, Gide 2019, Nathanson 2017 pre, Nathanson 2017 post, GSE78220) and one adoptive T cell therapy cohort (Lauss 2017 cohorts) in Melanoma: In each graph, the upper half depicted the survival analysis within the IL4I group, while the lower half illustrated the proportion of immunotherapy responses in the high and low IL4I1 groups.
