# Supplementary figures and images for: IL4I1: a novel molecular biomarker represents an inflamed tumor microenvironment and precisely predicts the molecular subtype and immunotherapy response of bladder cancer
Source: Front Pharmacol. 2024 May 30;15:1365683. doi: 10.3389/fphar.2024.1365683 (PMC11169701; doi:10.3389/fphar.2024.1365683)

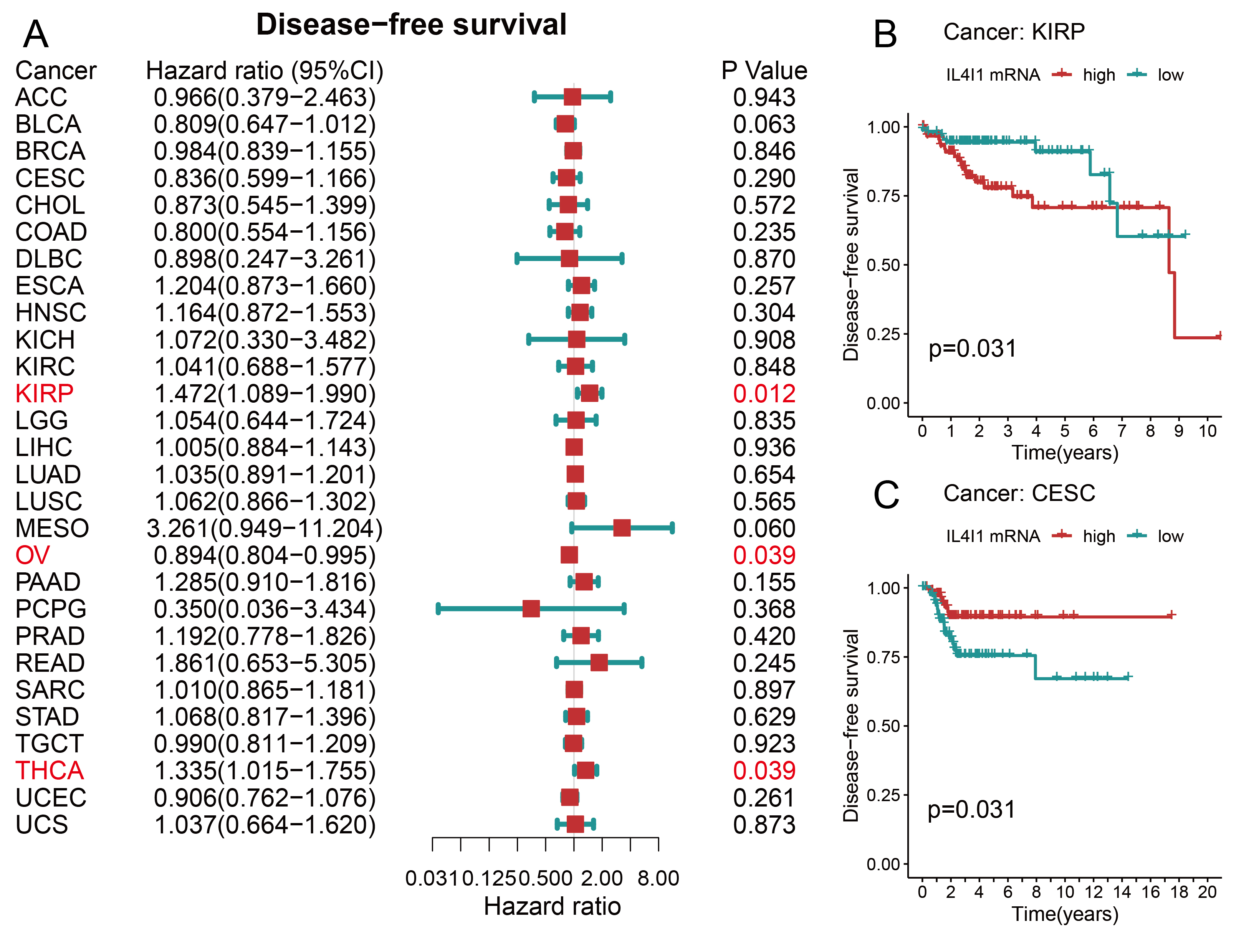

Supplement: Supplementary file 1 [file Image3.JPEG]

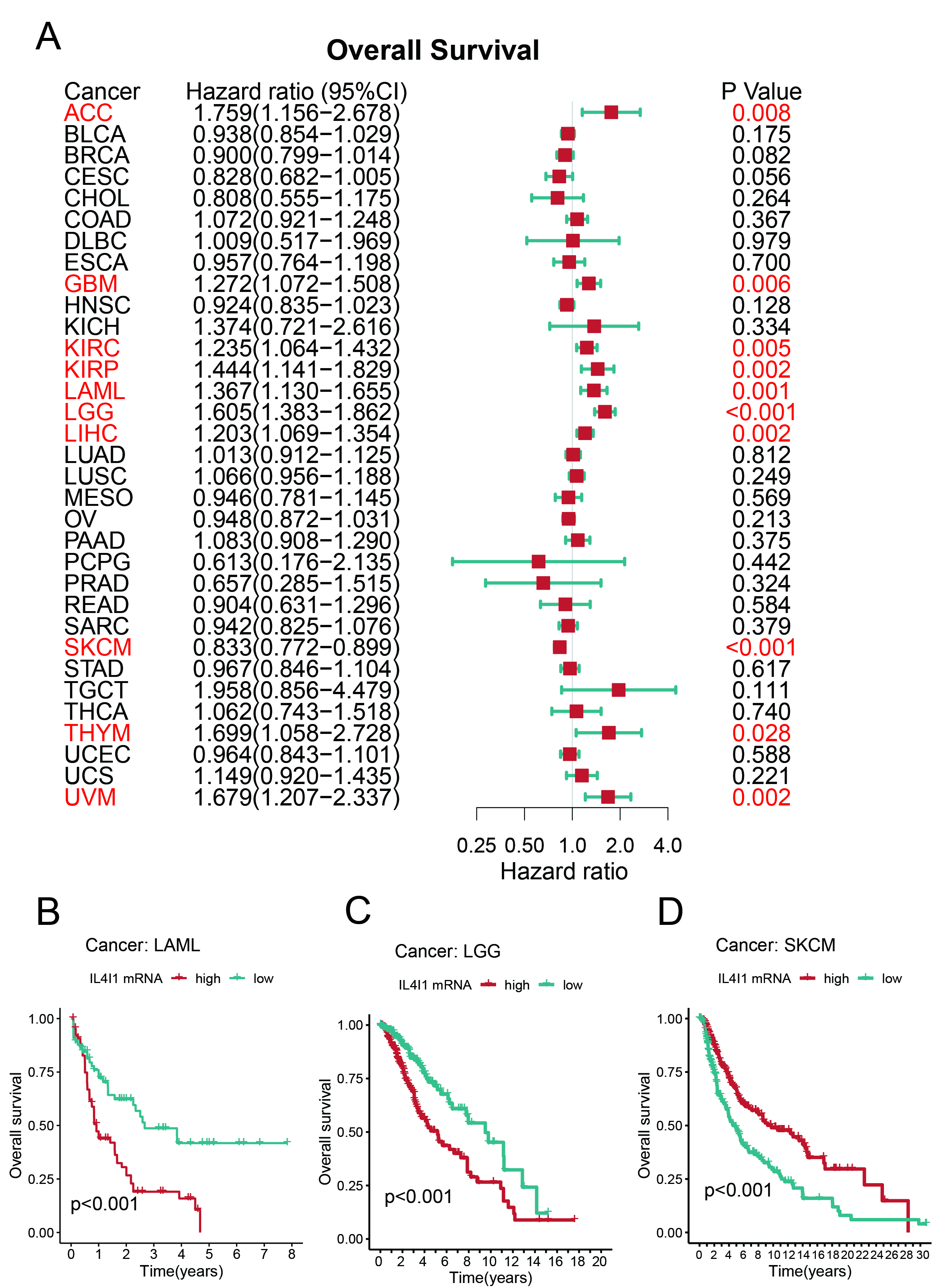

Supplement: Supplementary file 2 [file Image1.JPEG]

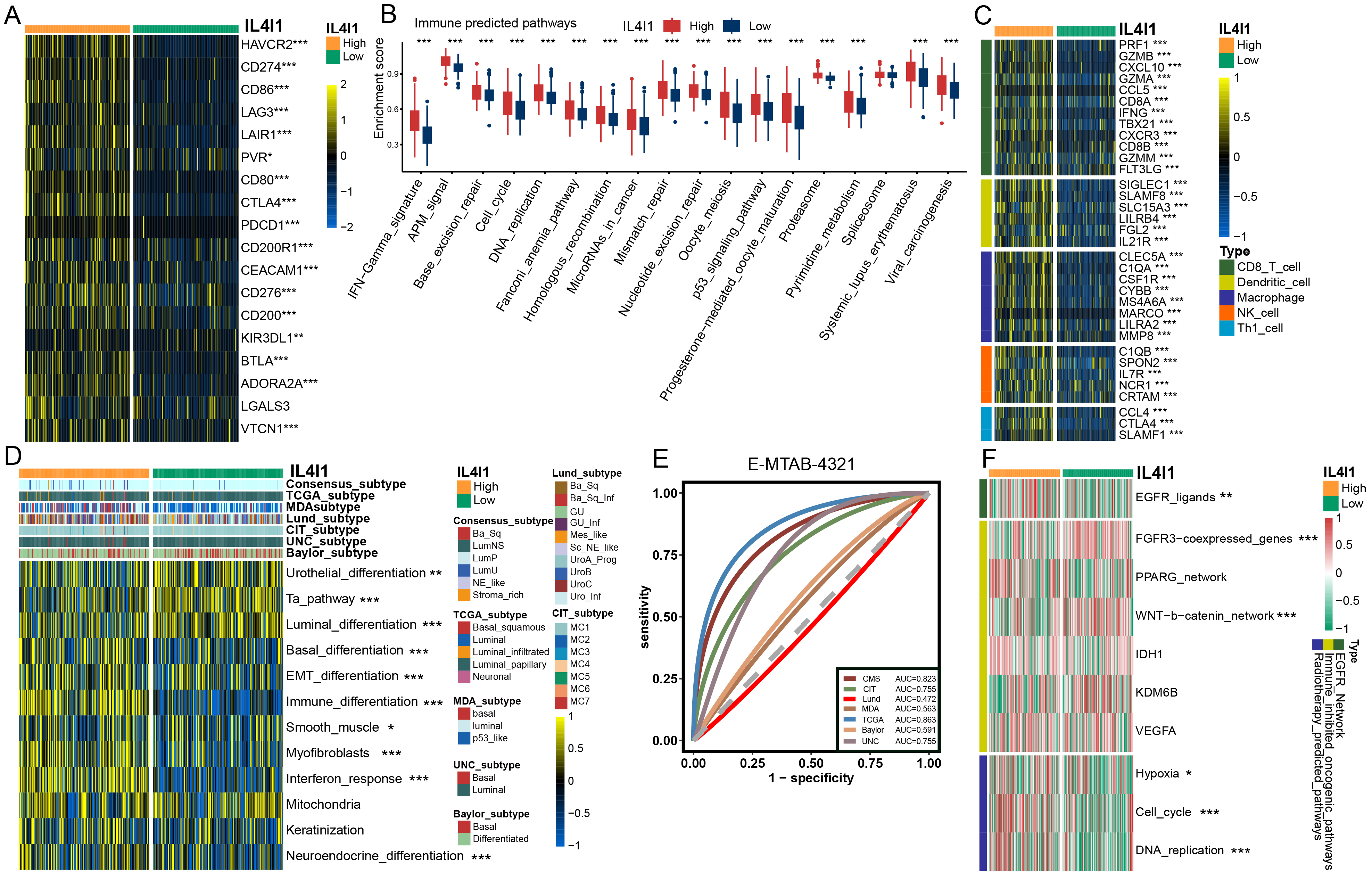

Supplement: Supplementary file 3 [file Image4.JPEG]

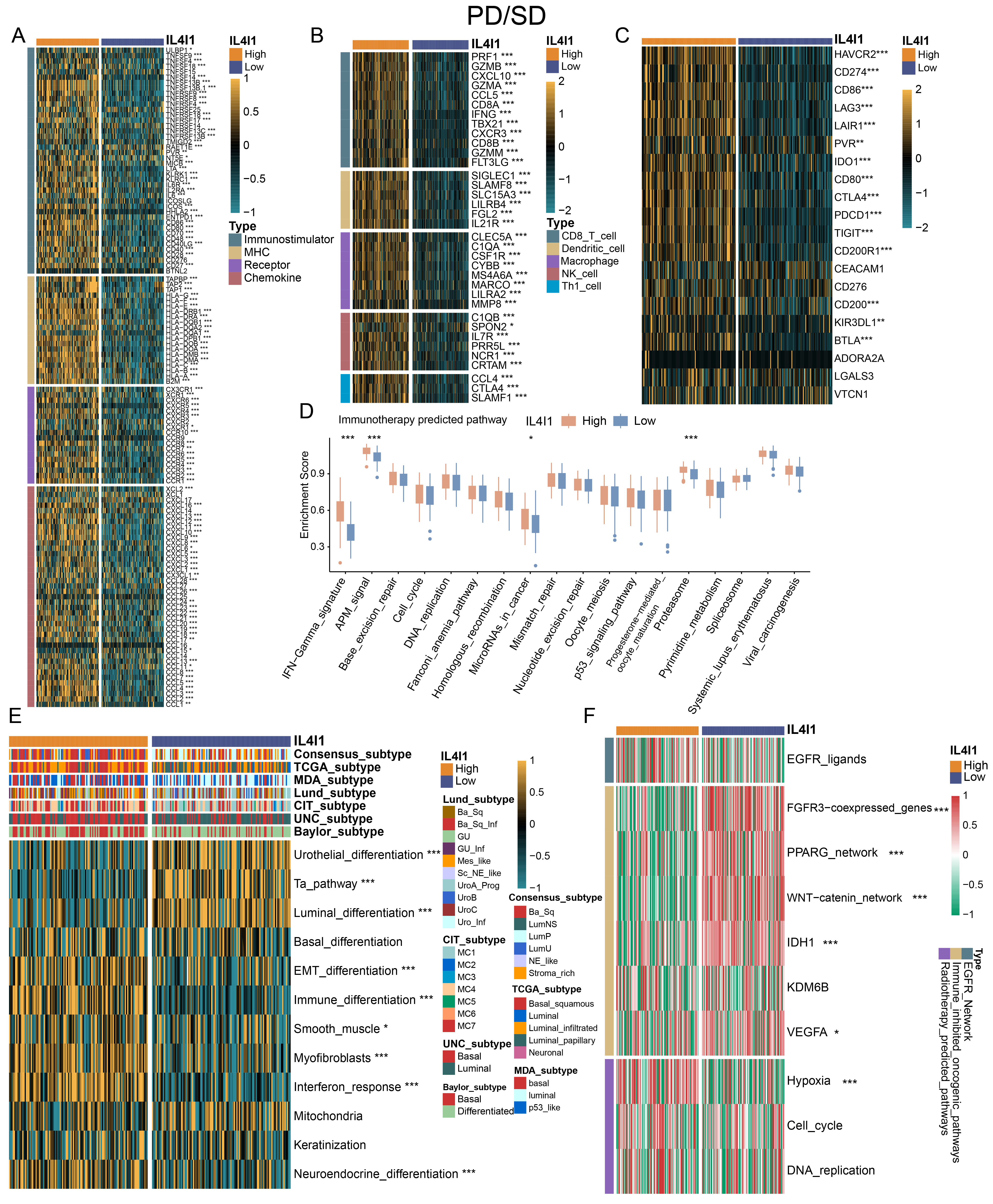

Supplement: Supplementary file 4 [file Image7.JPEG]

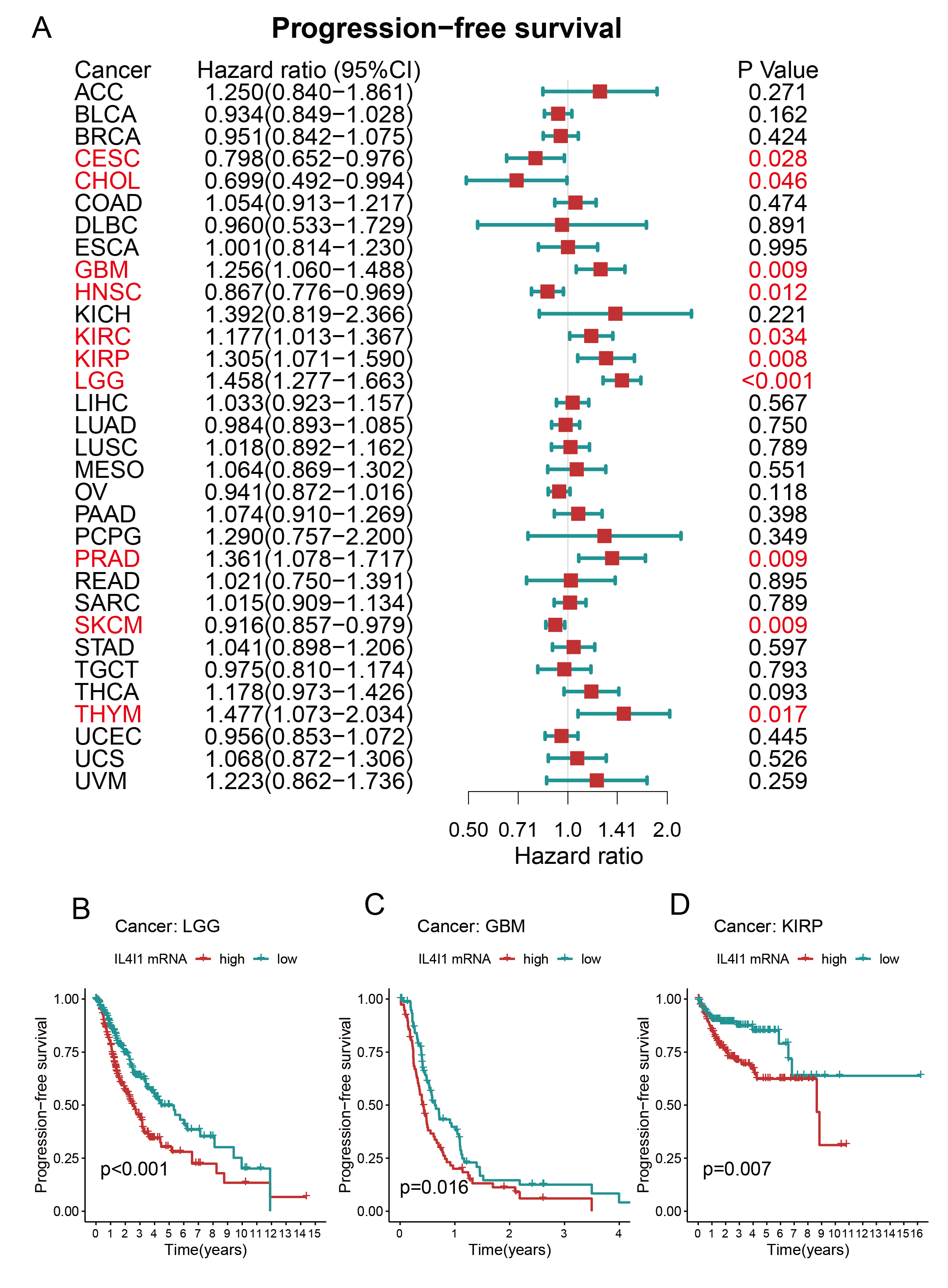

Supplement: Supplementary file 5 [file Image2.JPEG]

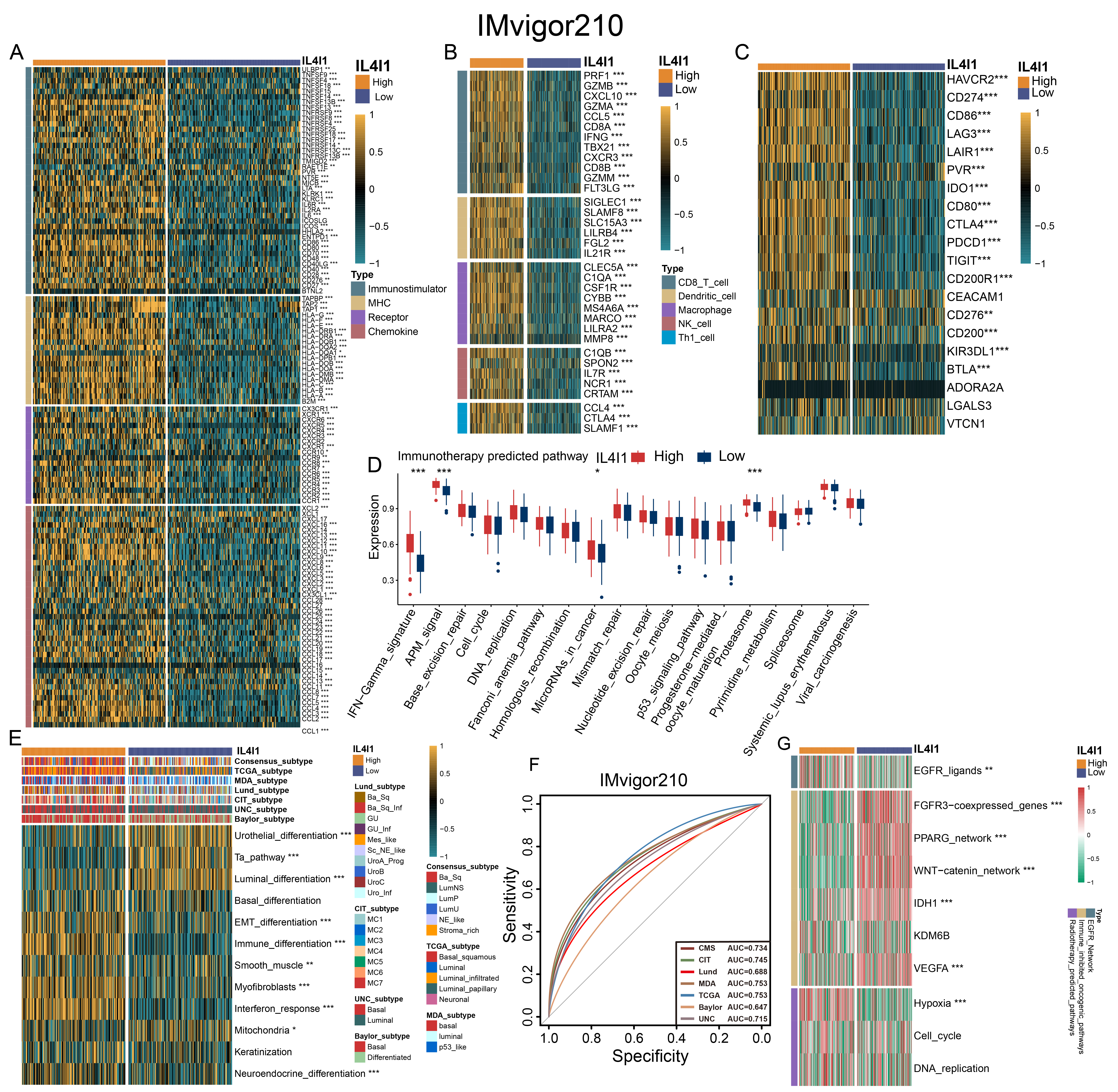

Supplement: Supplementary file 6 [file Image5.JPEG]

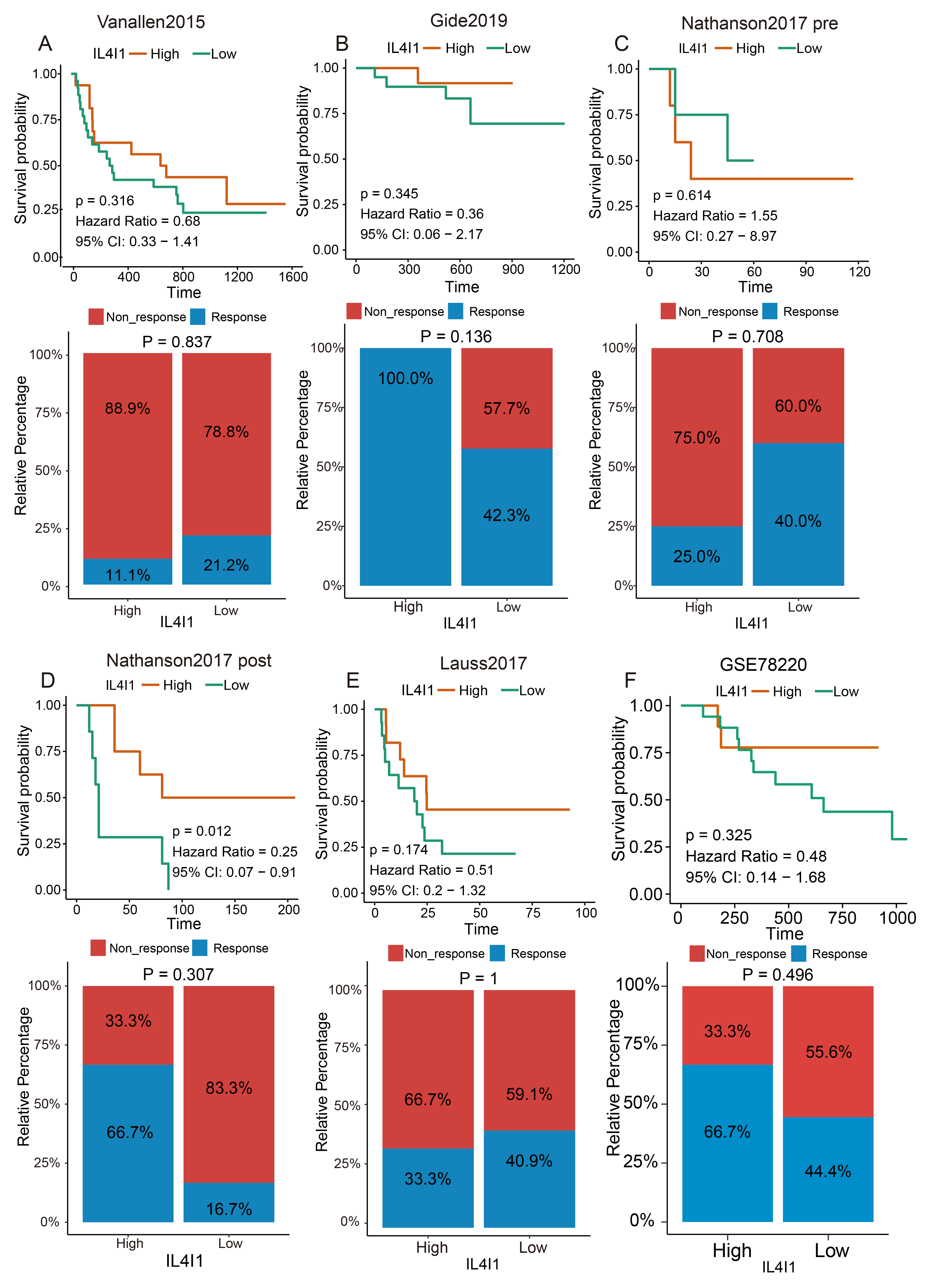

Supplement: Supplementary file 7 [file Image8.JPEG]

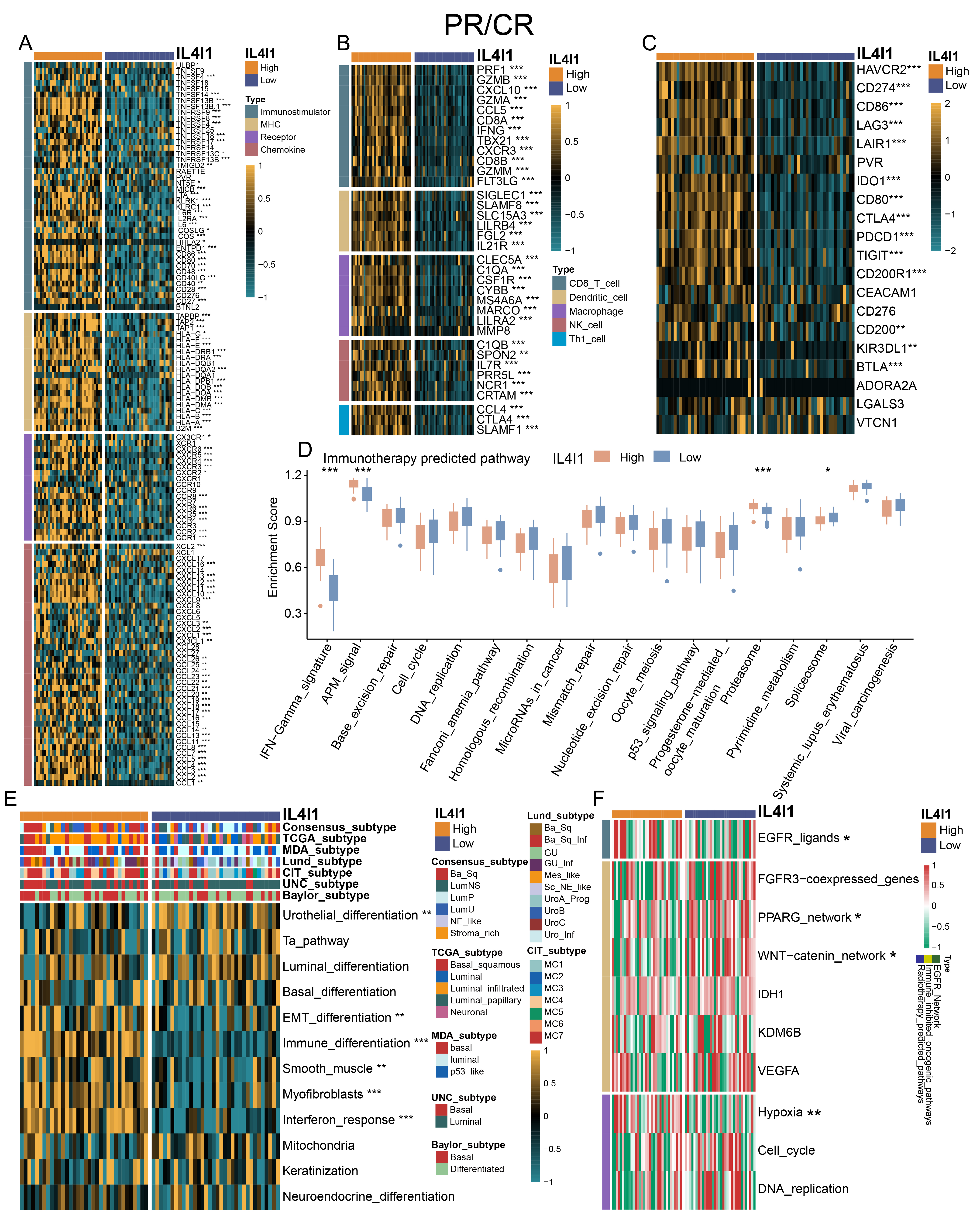

Supplement: Supplementary file 8 [file Image6.JPEG]
